# Meeting abstracts from the 12th European Conference on Rare Diseases and Orphan Products

**DOI:** 10.1186/s13023-024-03293-9

**Published:** 2024-12-05

## S1 The Syndrome Without a Name clinic (SWAN) Wales

### Graham Shortland, Zoe Morrison, Matthew Spencer

#### Department of Child Health, Cardiff University Health Board, Cardiff, UK

***Orphanet Journal of Rare Diseases*** 2024, 18(1): S1

**Background:** The SWAN Clinic is the only commissioned type of clinic of its kind within the UK. Currently, when a child or adult has exhausted all routine NHS testing routes, they remain without a diagnosis. The SWAN Clinic offers our Welsh resident population, with a suspected rare disease an alternative pathway for diagnosis.

**Results:** Over two years (2022-2024) 70 paediatric patients and 80 adult patients suspected of having a rare disease have been referred.

A diagnosis has been provided to 8% of all adult and paediatric patients seen by the clinic. In those patients who have completed investigation and been discharged, the diagnostic rate rises to 35%.

The SWAN clinic has participated in a self-evaluation since with the collection of patient reported outcome measures (PROMs) and patient reported experience measures (PREMs), collected at a patient’s pre-clinic visit, 6-months after their SWAN clinic appointment and at discharge. The PROM captures the patient/family’s Self-Management Assessment Scale (SMASc) and their Perceived Personal Control. Through the care coordination provided by the SWAN Clinic model we have demonstrated an improvement in all 5 of the domains of the SMASc across a 6-month period.

We have demonstrated a statistically significant reduction in secondary care healthcare utilisation. The general population in Wales will have an average of 1 contact day with secondary health care across a 6-month period (outpatient appointments, inpatient admission days and A&E attendances). When compared with the SWAN clinic population prior to first contact, this it is dramatically higher, an average of 18. After 6-months of being under the SWAN Clinic, this reduces to 8, demonstrating that our care model can reduce secondary healthcare utilisation by 56%.

**Conclusions:** A new clinical approach for patients suspected of having a rare disease not yet diagnosed has been developed as a pilot project in Wales. Diagnoses have been made in previously undiagnosed patients which have resulted in important changes to care, including treatment, management and necessary referrals in a significant number of adult and paediatric patients. We have provided information, not previously available to families, enabling them to make reproductive choices.

Through using PREMs, PROMS and patient interviews we have demonstrated improvements to patient experience particularly in care co-ordination. We have shown potential benefits to a reduction in healthcare utilisation as a consequence of this service.

**Acknowledgements**

The staff and patients of the SWAN (Wales) Service.

## S2 Early genome sequencing in patient care

### Miriam Elbracht*, Ingo Kurth*

#### Institute for Human Genetics and Genomic Medicine, Uniklinik RWTH Aachen, Medical Faculty, Aachen, Germany

^*^Corresponding author: mielbracht@ukaachen.de; ikurth@ukaachen.de.

***Orphanet Journal of Rare Diseases*** 2024, 18(1): S2

**Background:** 80% of all rare diseases and at least 10% of all cancers are caused by a germline variant [1]. The total number of genetic diseases therefore accounts for a substantial proportion of all diseases worldwide. It is becoming increasingly clear that accurate molecular diagnosis is the basis for correct management and treatment [2, 3]. A lack of diagnosis leads to false treatment, incorrect and late prevention or intervention and has a negative impact on the outcome of the patient and their families [4].

Despite new technical possibilities, however, genetic testing is often carried out rather late and too hesitantly, and there is still little awareness even among doctors that genetic diseases occur in all subdisciplines of medicine [4].

**Materials and methods:** We performed genome sequencing (GS) in a heterogeneous cohort of 350 index patients with suspected monogenic diseases from various medical disciplines at a maximum-care university hospital. The healthy parents were preferentially included in the analysis (trio analysis), so that >1000 short-read genomes were sequenced. The sequencing was embedded in a genetic counselling before and after the genome analysis and accompanied by an anonymous questionnaire on the practicability of GS and the handling of the results. The recruitment phase has been completed and the preliminary results are being summarized.

**Results:** Depending on the indication, 20-60% of cases could be solved by GS. The highest rates of solved cases were found in syndromic, neurological and eye diseases. The survey showed that a total of 72% of patients were burdened or very burdened by the lack of diagnosis before the GS. 99% were very satisfied with the course of the study, 46% of the participants wanted to take the results into account in terms of family planning and 67% now saw the possibility of being able to make well-directed decisions regarding their future medical care after the GS.

**Conclusions:** GS allows a rapid diagnosis and thus a more targeted and early possibility of general and adapted therapy management and care. The majority of patients find the process of GS very practicable, the setting of GS being embedded in genetic counselling as very helpful and important and the majority of patients see a benefit from GS, even in the case of failure to diagnose through testing.

**Acknowledgements**

The study was partially supported (with reagents) by Illumina, Inc. The study was completed on behalf of the genoMED study group^#^.

^#^Matthias Begemann, Katja Eggermann, Thomas Eggermann, Nergis Güzel, Cordula Knopp, Florian Kraft, Jeremias Krause, Eva Lausberg, Annette Lischka, Larissa Mattern, Robert Meyer, Julia Suh

**Affiliation:** Institute for Human Genetics and Genomic Medicine, Uniklinik RWTH Aachen, Medical Faculty, Aachen, Germany

**References**

- [1] Nguengang Wakap, S., et al., *Estimating cumulative point prevalence of rare diseases: analysis of the Orphanet database.* Eur J Hum Genet, 2020. **28**(2): p. 165-173.
- [2] Lindstrand, A., et al., *Genome sequencing is a sensitive first-line test to diagnose individuals with intellectual disability.* Genet Med, 2022. **24**(11): p. 2296-2307.
- [3] Bick, D., et al., *Case for genome sequencing in infants and children with rare, undiagnosed or genetic diseases.* J Med Genet, 2019. **56**(12): p. 783-791.
- [4] Faye, F., et al., *Time to diagnosis and determinants of diagnostic delays of people living with a rare disease: results of a Rare Barometer retrospective patient survey.* Eur J Hum Genet, 2024.

## S3 

### Dorica Dan^1^*, Maria Puiu^2^, Emilia Severin^3^, Lidia Onofrei^4^, Adela Chiriță Emandi^5^, Ioana Streața^6^, Alexandra- Loredana Dan^7^

#### ^1^Romanian National Alliance for Rare Diseases - RONARD, NoRo Center for RD. ^2^Victor Babes University of Medicine and Pharmacy-Timisoara. ^3^Carol Davila University of Medicine and Pharmacy-Bucharest. ^4^Romanian Ministry of Health. ^5^Victor Babes University of Medicine and Pharmacy-Timisoara. ^6^University of Medicine and Pharmacy-Craiova. ^7^NoRo Center for RD

^*^Corresponding author: dorica.dan@eurordis.org

***Orphanet Journal of Rare Diseases*** 2024, 18(1): S3

**Background:** It was the second time RONARD organized a side event at ECRD. The goal of the ECRD satellite meeting was to inform and exchange the views of different stakeholders about the link between the national policies on RD and the EU policy framework for RD and to raise awareness around the collaborative efforts that must be done for clear policy recommendations that can influence both EU and national policies.

**Methods:** We organized the ECRD 2024 side event online on 13.05.2024 with 45 participants: patients, experts, patient organizations, centers of expertise, the president of EURORDIS, but also representatives from the Romanian national authorities, like Romanian Presidency, Minister of Health, National Agency for Medicines and Medical Technologies, Romanian MEPs and the Association for Innovative Drug Producers.

**Results:** The main challenges identified in the care system in Romania are: no national registry for rare diseases, no ORPHA code implemented (just ICD10 which don’t cover most of the diagnosis), still no proper care infrastructure, no financial support for CoE or Patient Organizations, a long waiting time from approval of an Orphan Drug by EMA and the actual access for patients in Romania, no national program for genetic testing for adults with rare diseases and not always good communication between medical and social services, and other stakeholders.

The main opportunities mentioned by the participants**:** NPRD is updated and integrated in the National Strategy for Health 2023–2030 with funding, there are funds available in the PNRR - National Program for Recovery and Resilience, National Operational Program for Health allocates funding for Centers of Expertise and Regional Network of Genetic Centers. Social services will be developed through POIDS - Operational Program Social Inclusion and Dignity. Another opportunity mentioned by all is the good collaboration between RONARD and stakeholders involved in Romanian care ecosystem, EU collaboration with Eurordis and ERNs, including participation in JARDIN.

**Conclusions:** There is a close link between the national policy on RD and the EU policy framework for RD. The next steps at national level: to organize a meeting at the Ministry of Health, monitor the implementation of the 3 priorities presented by the Minister of Health as priorities for RDs in 2024: upscaling of NBS, national registry and National Coordination Hub and present the results at the RD School for Journalists 2024 and Europlan conference, organize actions for upscaling of NBS, and finalize the Rare Diseases Law in 2024.

## S4 No health without mental health! Let's co-create a mentally healthy toolkit: areas of need experienced and research gaps

### Kirsten Johnson^1^*, Matt Bolz-Johnson^2^

#### ^1^Board Member, EURORDIS, Paris, France; Orcid ID 0000-0003-3733-4947. ^2^Mental Health Lead and Healthcare Advisor, EURORDIS, Paris, France.

*Corresponding author: kirsten.johnson@eurordis.com

***Orphanet Journal of Rare Diseases*** 2024, 18(1): S4

**Abstract**

The ‘No Health Without Mental Health! Let's Co-create a Mentally Healthy Toolkit’ session of the European Conference of Rare Diseases in Brussels, 15 May 2024, allowed participants to identify needs and propose domains for the toolkit. The toolkit should address commonalities experienced by the community, as well as be flexible and adaptive to condition-specific needs, able to address the diverse range of needs of children, young people and adults living with a rare condition and their wider family.

The proposed domains are:

1. **Self-care and support** for where there are no existing patient groups.
2. **Therapeutic community support** including the role and value of disease-specific patient groups, access to trusted information, family weekends, etc.
3. **Peer-to-peer support** on an individual level including mentoring, individually tailored information and support etc.
4. **Psychosocial care advocacy tools** to secure access where there is a service gap.
5. **Health and social care professional tools** and approaches.

Discussion revealed the high psychological impact of living with a rare condition and the areas of need experienced. As much of this is hearsay and lived experience, there was recognition of the need for further research. The next steps will include a perspective paper which will set out the impact and challenges of addressing unmet mental health needs of people living with rare conditions. Session participants highlighted the need to raise awareness and educate clinicians and medical professionals about the impact of rare conditions on mental health. Research on physical health overshadows the development of research in mental health, therefore this perspective article will explore the challenges and opportunities in performing research on the impact of rare conditions on mental health; and on QoL tools, PROMs and PREMs used in performing trials.

Further workshops will continue the co-creation process of creating a Mentally Healthy Toolkit and all participants were encouraged to continue to engage with these opportunities.

**Acknowledgements**

With thanks to Concha Mayo, Mental Health & Wellbeing Engagement Manager, EURORDIS, for her part in organising this session, and to all session participants and panelists for their ideas and engagement.

## S5 

### Kirsten Johnson^1^*, Matt Bolz-Johnson^2^

#### ^1^Board Member, EURORDIS, Paris, France; Orcid ID 0000-0003-3733-4947. ^2^Mental Health Lead and Healthcare Advisor, EURORDIS, Paris, France

*Corresponding author: kirsten.johnson@eurordis.com

***Orphanet Journal of Rare Diseases*** 2024, 18(1): S5

**Abstract**

As part of the European Conference of Rare Diseases, an audience of researchers, experts by lived experience, clinicians and industry joined together to begin the co-creation process for making a Mentally Healthy Toolkit in the session entitled ‘No Health Without Mental Health! Let's Co-create a Mentally Healthy Toolkit’. The first part of the session allowed participants to express the population needs via Mentimeter polls, questions from the floor and panel discussion. This identified the common needs faced by those living with a rare condition and explored the existing approaches and tools, learning how they have worked and testing if they are applicable to form the basis for the new toolkit.

The participants highlighted four common needs: 1. Living with uncertainty and coping with change, grief and loss; 2. Reducing isolation and connecting with others; 3. Confidence to speak up and breaking down stigma; and 4. Self-care strategies. The toolkit will be developed specifically to address the common unmet mental health needs experienced by the rare disease community.

There is already published a systematic review on the importance of psychological support at diagnosis [1]. There will now be a systematic literature review to gather evidence on psychological support when living with a rare condition after the diagnosis. This new review will included aspects of the:

- Impact of rare conditions that cause social isolation and reduced sense of belonging because of chronic health condition and/or impact of visible or invisible differences.
- Challenge of living with chronic uncertainty over time.
- Impact of living with a rare condition on the family relationship (parents as a couple and siblings).

The systematic review will evidence the need of this toolkit, and further define the aspects which should be covered by the Mentally Health Toolkit. The co-creation process will continue with workshops on each of the themes identified, based on evidence and drawing on the manifold experiences of those living with rare conditions.

**Acknowledgements**

With thanks to Concha Mayo, Mental Health & Wellbeing Engagement Manager, EURORDIS, for her part in organising this session, and to all session participants and panellists, for their ideas and engagement.

**References**

- Kenny T, Bogart K, Freedman A, Garthwaite C, Henley SMD, Bolz-Johnson M, Mohammed S, Walton J, Winter K, Woodman D. The importance of psychological support for parents and caregivers of children with a rare disease at diagnosis. Rare Disease and Orphan Drugs Journal. 2022; 1(2): 7. http://dx.doi.org/10.20517/rdodj.2022.04

## S6 Collaborating for change: transforming rare disease research and outcomes through public-private partnerships

### Magda Chlebus^1,2^, Sheela Upadhyaya^3^, Danielle Dong^4^, Kira Gillett^5^, Roseline Favresse^6^, Matt Bolz-Johnson^6^, Alexandre Bétourné^7^, Salah-Dine Chibout^8^, Holm Graessner^9^, Clara Romero^10,3^, Mathieu Boudes^2,3,11^*

#### ^1^European Federation of Pharmaceutical Industries and Associations, Brussels, Belgium. ^2^Rare Disease Moonshot, Brussels, Belgium. ^3^Together For Rare Disease (T4RD), Brussels, Belgium. ^4^Sanofi, Cambridge, Massachusetts, USA. ^5^The AMP© Bespoke Gene Therapy Consortium, Foundation for the National Institutes of Health, North Bethesda, Maryland, USA. ^6^EURORDIS-Rare Diseases Europe, Paris, France. ^7^Critical Path Institute (C-Path), Tucson, Arizona, USA. ^8^Novartis Pharma, Basel, Switzerland. ^9^Centre for Rare Diseases, University Hospital Tübingen, Tübingen, Germany. ^10^FIPRA, Brussels, Belgium. ^11^Montsouris Consilium, Montpellier, France

*Corresponding author: mathieu.boudes@montsourisconsilium.fr

***Orphanet Journal of Rare Diseases*** 2024, 18(1): S6

**Background:** Despite substantial investments in rare disease research, only 5% have approved treatments. Public-Private Partnerships (PPPs) represent a promising approach to enhance innovation and accelerate therapy development. Politically, there is a growing recognition of the need for stronger partnerships to tackle the challenges in rare disease research and therapy development.

**Results:** Public-private partnerships (PPPs) are crucial for promoting innovation into precision therapy in healthcare, with the example of the AMP© Bespoke Gene Therapy Consortium aiming to streamline the navigation of the regulatory process with the Regulatory Playbook and enhance the accessibility of adeno-associated virus (AAV) technology. The Rare Disease Cures Accelerator-Data and Analytics Platform (RDCA-DAP), developed by the Critical Path Institute, demonstrated the importance of standardizing data collection methods and creating centralized platforms to facilitate more efficient regulatory processes and therapy development. It has significant engagement from academia, industry, and regulatory agencies, with approximately 400 users accessing and analyzing the data. The Innovative Health Initiative (IHI) is another successful PPP model, promoting collaboration between industry, academia, and patient organizations to accelerate the development of innovative medicines, solutions, and research infrastructures. The importance of co-creation, transparent communication, and mutual goals for successful partnerships was emphasized, for e.g. the Sanofi-ERN BOND collaboration showed how early stakeholder engagement leads to impactful solutions and effectively addresses unmet needs in research. Pre-competitive collaboration was recognized as a significant opportunity to enhance the collaborative spirit within PPPs and eliminate competitiveness. The session also stressed the role of the human factor to foster trust and establish robust foundations to establishing a unified project scope, defining research questions, and building personal relationships among partners, particularly in the early stages of a project.

**Conclusions and next steps:** Strengthening PPPs is essential for advancing therapy development for rare diseases. Enhanced collaboration, effective co-creation, transparent communication, and standardized data collection are crucial for driving innovation. Key actions include advocating for new partnership models, removing barriers, involving patients and their representatives, focusing on incremental advancements, and sustaining pre-competitive collaborations. By implementing these steps, Europe can enhance its research capabilities and expedite treatment development for rare diseases.

**Acknowledgments**

Both multi-stakeholder initiatives, Rare Disease Moonshot sets out strategic goals to address research gaps affecting people living with a rare disease without solutions and Together for Rare Diseases aims at supporting European Reference Networks (ERNs) to collaborate with pharmaceutical industry to pursue opportunities that will address unmet medical needs of people living with rare diseases. The authors thank the RD Moonshot and Together4RareDiseases for jointly organising this insightful session.

## P1 A Rare Find - newborn screening collaborative

### John Lee Taggart^1^, Bob Stevens^2^, Toni Mathieson^1^, Georgina Morton^3^

#### ^1^Niemann-Pick UK (NPUK), Washington, UK. ^2^MPS Society, Amersham, UK. ^3^Archangel MLD Trust, London, UK

***Orphanet Journal of Rare Diseases*** 2024, 18(1): P1

A Rare Find is a comedy short film with a difference...as it hopes to raise awareness and start conversation surrounding newborn screening, the blood spot/heel prick test*, and the desperate need for positive progress within the current UK newborn screening programme (which at present, screens for only 9 conditions, for context the USA screen for up to 59!)

Life with a newborn is hard enough, as our struggling young parents find, but the challenges of a long “diagnostic odyssey” can make this even harder. Early screening can be a huge positive step to find out if a rare condition is present, as early intervention and therapy is absolutely key.

This is why we have brought together multiple rare disease groups in the UK to push collectively for progress...the film, poster and wider campaign will help to achieve this. All we want is to "test early, to treat early - let's help families thrive!"

The short won Gold at the Smiley Charity Film Awards 2024, was nominated for an RTS North East & Borders 2024 award and was an official selection at the World Health Organization’s Health4All 2024 festival. It currently has achieved approximately 1 million views on the official Smiley TikTok account.

## P2 Streamlining second opinions for rare diseases in Brazil: an evidence-based digital platform engaging key stakeholders

### Vinícius Lima^1,2,^*, Filipe Bernardi^1,2^, Diego Yamada^1^, João Baiochi^1^, Victor Cassão^1^, Ida Schwartz^2,3^, Têmis Félix^2,3^, Victor Ferraz^1^, Domingos Alves^1,2^

#### ^1^Ribeirão Preto Medical School, University of Sao Paulo, Ribeirão Preto, Brazil. ^2^National Institute of Rare Diseases, Porto Alegre Clinical Hospital, Porto Alegre, Brazil. ^3^Medical Genetics Service, Porto Alegre Clinical Hospital, Porto Alegre, Brazil

*Corresponding author: viniciuslima@alumni.usp.br

***Orphanet Journal of Rare Diseases*** 2024, 18(1): P2

In the challenging landscape of rare diseases, healthcare practitioners and patients often grapple with the complexities of accurate and timely diagnoses [1]. This complexity is primarily due to limited evidence, the rarity of specialized resources, the potential lack of adequate human and technological resources, and the intricacies inherent to such conditions [2]. Therefore, the second opinion represents an essential resource for assertive clinical practices.

Information and communication technologies can expand the health care offer and reach. As the world increasingly adopts these technologies, healthcare stands to gain immensely. Harnessing this potential, we introduce an innovative Second Opinion platform tailored for rare diseases. Designed through a sociotechnical lens and adhering to data protection regulations, this platform seamlessly integrates the collective knowledge of researchers, medical specialists' expertise, health professionals' experience, and patient associations' invaluable perspectives, making them key stakeholders in this initiative. Figure 1 illustrates the Second Opinion for the Rare Diseases Platform.

**Figure 1:**
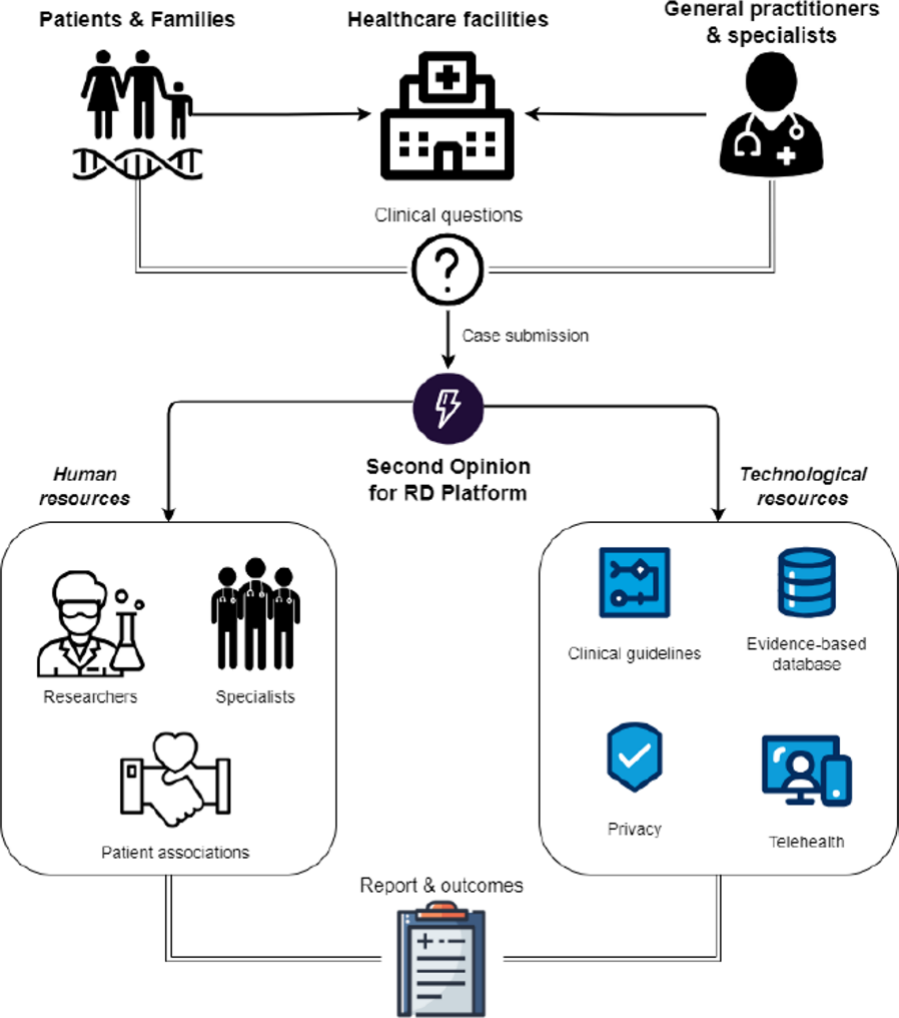
Overview of the second opinion for the rare diseases platform

The primary objective of this project is to fundamentally streamline the process of obtaining specialized second opinions on rare diseases. It seeks to anchor these opinions firmly within rigorous scientific and clinical evidence. The platform ensures flexibility in communication by providing both asynchronous and synchronous channels for medical discussions. Whether it is a real-time consultation between a general practitioner in a remote area and a specialist in a metropolitan hospital or a forum-based discussion analyzing the intricacies of a specific case, the platform facilitates it all.

However, its aspirations go beyond mere consultations. Recognizing the importance of public education, the platform is also geared towards building an extensive public educational knowledge base for formative second opinions. These repositories, designed to be informative and user-friendly, play a crucial role in informing and augmenting the decision-making processes, both for healthcare professionals and patients.

In conclusion, this initiative envisions a future where the accessibility gap is bridged, patients have better and quicker access to specialized care, and healthcare professionals are empowered with a wealth of knowledge and collaborative tools at their fingertips.

**Acknowledgments**

This study is supported by the National Council for Scientific and Technological Development (CNPq) - Grant No. 406857/2022-9, and by the São Paulo Research Foundation (FAPESP) - Grant No. 2023/10203-8. The project is also endorsed by the Brazilian National Rare Disease Network (http://raras.org.br/).

**References**

- [1] Marwaha S, Knowles JW, Ashley EA. A guide for the diagnosis of rare and undiagnosed disease: beyond the exome. Genome Med. 2022;14:23. Available at: https://doi.org/10.1186/s13073-022-01026-w
- [2] Hayeems RZ, Michaels-Igbokwe C, Venkataramanan V, Hartley T, Acker M, Gillespie M, et al. The complexity of diagnosing rare disease: An organizing framework for outcomes research and health economics based on real-world evidence. Genet Med. 2022;24(3):694-702. Available at: https://doi.org/10.1016/j.gim.2021.11.005

## P3 Case management for rare diseases in Romania

### Dorica Dan^1,^*, Nicoll Andreescu^2^, Alexandra Dan^1^, Emilia Severin^3^, Maria Puiu^2^, Lidia Onofrei^2,4^

#### ^1^Romanian National Alliance for Rare Diseases, NoRo Center for RD. ^2^Victor Babes University of Medicine and Pharmacy – Timișoara. ^3^Carol Davila University of Medicine and Pharmacy-Bucharest. ^4^Romanian Ministry of Health

^*^Corresponding author: dorica.dan@eurordis.org

***Orphanet Journal of Rare Diseases*** 2024, 18(1): P3

**Background:** The problem we are addressing is the diagnosing and access to proper care for patients with rare diseases in Romania. 90% of them are still undiagnosed and struggle to access the services they need, 50% are children, and 65% of patients must visit at least 7 different physicians before diagnosis. Approximately 50 % of the Romanian population lives in rural areas, with no access to care services. Care pathways for rare diseases are not structured and patients don’t know where to go. Rare diseases patients are invisible for the care system in many cases because our country uses ICD10 and not ORPHA codes and have not a national registry for rare diseases.

**Methods:** We were partners in INNOVCare project during 2015–2018 and piloted case management for rare diseases in our region. We have identified that community nurses have the background and could support even the most isolated patients living with rare diseases in Romania [1]. RPWA has addressed the Ministry of Health to include the case management in the law, as a working method for people living with rare diseases, included case management in their job description and signed a partnership for training them in case management for rare diseases and support the care coordination for patients with rare diseases. The partnership has been signed in April 2021. 850 community nurses out of 1850 have been trained up to date, through our projects and contributed to other trainings on specialization of 300 community nurses. The methodology used in our training is combined, face to face and online, using ECHO-Extension of Community Health Outcomes (online, based on case studies, where all teach, and all learn), bringing in our trainings experts from the accredited Centers of Expertise and provided extra tools to help their work: an online map of services in Romania and a virtual case management tool [2,3].

**Results:** We conducted a survey to assess the importance of the knowledge gained by community nurses in our training. 17.51% of the trained community nurses declared that they have included new patients with RD in case management already during the training, 95,6% declared that the information provided in the training was very useful for their activity and 92.6 % considered that coordinating patients to Centers of Expertise is extremely useful.

**Conclusions:** Through our multi-stakeholder collaboration at national level, we are reducing the waiting time for diagnosis and care for rare diseases patients.

**Acknowledgements**

We extend our sincere gratitude to the National Network of Community Healthcare in Romania for their invaluable support and collaboration in the implementation of this project. Their contribution has been essential in improving the care for patients with rare diseases in Romania.

**References**

- [1] Bridging the gaps between health, social and local services, to improve care for people living with rare and complex conditions: key findings of the EU-funded INNOVCare project and its case management pilot, August 2019, International Journal of Integrated Care 19(4):566 - https://www.ijic.org/articles/abstract/10.5334/ijic.s3566/
- [2] Shared Values and Vision most important of nine pillars of Integrated Care https://healthmanager.ie/2022/03/shared-values-and-vision-most-important-of-nine-pillars-of-integrated-care/
- Nine Pillars of Integrated Care - IFIC (integratedcarefoundation.org).

## P4 Innovations in therapy development for rare diseases through the Rare Disease Cures Accelerator – Data and Analytics Platform

### Heidi Grabenstatter, Dominique Cruz, Laura Hopkins, Ramona Walls, Collin Hovinga, Alexandre Bétourné

#### Critical Path Institute, Tucson, Arizona, USA

***Orphanet Journal of Rare Diseases*** 2024, 18(1): P4

Progress toward the development of therapies for rare diseases is hampered by a limited biological understanding of most diseases and their progression and challenges in evaluating patients and designing adequate clinical trials. Data that could be used to characterize many diseases is often collected in multiple formats, including electronic health records, patient-report registries, clinical natural history studies, and past clinical trials. However, each data source contains a limited number of subjects and different data elements, and is frequently kept proprietary in the hands of the study sponsor. The Rare Disease Cures Accelerator – Data and Analytics Platform (RDCA-DAP®) is an FDA-funded effort to overcome these persistent challenges. By aggregating data across all rare diseases and making that data available to the community to support understanding of rare diseases and inform drug development, RDCA-DAP aims to accelerate the regulatory approval of new therapies. RDCA-DAP curates, standardizes, and tags data across datasets to make it findable within the database, and contains a built-in analytics platform to help visualize, interpret, and use it to support drug development. RDCA-DAP also coordinates data and tool resources to serve a diverse array of rare disease stakeholders that includes academic researchers, drug developers, regulatory reviewers and patients and their caregivers. The platform currently holds over 70 international datasets for 35 different rare diseases, with an ever-expanding and diversifying database. The platform users range from industry and academia to foundations who are actively leveraging the database to inform their research and drug development programs. In addition, the RDCA-DAP team is developing data-driven solutions for several rare diseases under the premises of the RDCA-DAP Task Forces. Each Task Force convenes patient advocacy groups, foundations, key opinion leaders and industry in a pre-competitive environment to drive single deliverables aimed at disentangling bottlenecks in drug development and solving unmet needs in rare diseases. Our active Task Forces and collaborative groups include efforts for Friedreich’s Ataxia, Progressive Supranuclear Palsy, rare mitochondrial and inherited metabolic diseases and Limb-girdle muscular dystrophy. RDCA-DAP aims to develop a Task Force for each organ or bodily function impacted by rare diseases. Finally, we encourage rare diseases stakeholders to utilize the RDCA-DAP platform to develop solutions on their own and/or through collaboration with our program.

Platform access: https://portal.rdca.c-path.org/. Connect with our team: RDCA-DAPadmin@c-path.org

## P5 Preliminary results of the establishment of a Youth Advisory Council focused on drug development within pediatric nephrology

### Eva Degraeuwe^1,2^, Aline Vlaeminck^1^, Maria Cavaller^1,2^, Elke Gasthuys^1^, Ann Raes^1,2,3^, Johan Vande Walle^1,2^, Segolene Gaillard^4^, Begonya Nafria Escalera^5^, Evelien Snauwaert^1,2,3^

#### ^1^Departement of Internal Medicine and Pediatrics (GE35), Ghent University, Ghent, Belgium. ^2^Department of Pediatric Nephrology, Ghent University Hospital, Ghent, Belgium. ^3^ERN ERKNet. ^4^Hospices Civils de Lyon, Lyon, France. ^5^Sant Joan de Déu Children's Hospital, Barcelona, Spain

***Orphanet Journal of Rare Diseases*** 2024, 18(1): P5

**Background:** After the 2007 Pediatric Regulations, the number of pediatric clinical trials increased significantly. Many trials are still unsuccessful, partially related to poor study design, but mostly due to insufficient recruitment rates and low trial completion. Integrating patient feedback and experiences into the drug development cycle has been shown to enhance the design, the participant enrollment, and the retention in clinical trials for adults, ultimately leading to improved health outcomes and advantages for all parties involved. Despite these benefits, actively involving patients in the decision-making process (known as patient public involvement or PPI) is not yet a universally adopted practice. Additionally, there is a notable lack of such patient-informed data for clinical trials involving children. European countries such as France, Spain and Italy initiated pediatric PPI network. A novel and unique to Belgium Youth Advisory Council has been established to gather insights and commentary into clinical trials related to rare diseases and Pediatric Nephrology from a patient perspective.

**Method:** The Youth Advice council was founded in August 2023 at the Department of Pediatric Nephrology at Ghent University Hospital in Belgium. All sessions are being and will continue to be recorded and transcribed as part of our qualitative data collection process, which is scheduled for review in August 2024. Expert advice was consulted through eYPAG-Net on the design and the methodology of group sessions. The first session in October–November 2023 included education on drug development through clinical trials.

**Results:** A total of 10 pediatric participants with rare chronic kidney diseases were included. The parents were able to join the initial session, which was done in one case. Early results include language modification of the study ICF, improved communication to participants as well as a well-experienced education on clinical trials. The follow-up sessions will focus on the review of an industry-based informed consent through interview and questionnaire, as well as an open discussion on medical needs for development within Nephrology. A series of informal sessions have been added to strengthen group interaction over the coming months.

**Conclusion:** Aside from being widely agreed upon that PPI of pediatric patients and their families is important, the youth advice council in Pediatric Nephrology is a unique disease-specific PPI within Belgium. An expansion to other disciplines, as well as collaboration with other universities is apparent.

**References**

- [1] Cavaller-Bellaubi M, Faulkner SD, Teixeira B, Boudes M, Molero E, Brooke N, et al. Sustaining Meaningful Patient Engagement Across the Lifecycle of Medicines: A Roadmap for Action. Ther Innov Regul Sci [Internet]. 2021 Sep 1 [cited 2022 Mar 11];55(5):936–53. Available from: https://pubmed.ncbi.nlm.nih.gov/33970465/
- [2] Perfetto EM, Burke L, Oehrlein EM, Epstein RS. Patient-focused drug development a new direction for collaboration. Med Care. 2015 Jan 20;53(1):9–17.
- [3] J B, S S, C M, S H-M, J H, C T, et al. A systematic review of the impact of patient and public involvement on service users, researchers and communities. Patient [Internet]. 2014 Nov 22 [cited 2021 Oct 15];7(4):387–95. Available from: https://pubmed.ncbi.nlm.nih.gov/25034612/

## P6 Researching the lived experience of rare disease through participatory arts-based and creative methods

### Richard Gorman^1^*, Bobbie Farsides^2^

#### ^1^Research Fellow in Bioethics, Brighton and Sussex Medical School, Brighton, UK. ^2^Professor of Clinical and Biomedical Ethics, Brighton and Sussex Medical School, Brighton, UK.

*Corresponding author: r.gorman@bsms.ac.uk

***Orphanet Journal of Rare Diseases*** 2024, 18(1): P6

The Ethical Preparedness in Genomic Medicine (EPPiGEN) project examines how the promise and challenge of genomics is understood and experienced by those providing and engaging with genomic medicine services in the UK. It aims to produce a rich and nuanced account of the ethical issues resulting from moves to further embed and ‘mainstream’ genomic interventions within the nationalised healthcare system. Given the growing evidence that engagement with the creative arts can bring new insights to healthcare research [1], a major strand of EPPiGEN’s work has used artistic and creative approaches to collect stories from families affected by rare conditions.

We have worked in partnership with affected families to think imaginatively and critically about how their accounts of patient experience can be captured and used to help healthcare professionals better understand their perspectives. Our participant partners are parents of children with rare conditions, all of whom have interacted with genomic medicine services, many of whom have formal roles as patient representatives and/or advocates. This group has shaped our research throughout, guiding our development of questions, methods, and dissemination. Our aim has been to understand people’s hopes, expectations, and worries, and to provide an outlet for reflecting on what everyday life is like when much focuses on the promise of genomic medicine.

Participants have engaged with life-writing, stop-motion animation, poetry, collaging, and graphic medicine as creative ways of reflecting on their concerns, the challenges they face, and their significant moments and encounters [2–4]. A particular example is *Helix of Love*, a published collection of poetry written by parents of children with rare genetic conditions, which has received critical acclaim from stakeholders across the genomics sector, and has been embraced as a training resource in several countries. The work is recognised as offering invaluable insights to those planning and providing care for families affected by rare disease. The collection has allowed parents to share their experiences through channels, and on platforms, they would not have otherwise had access to. *Helix of Love* is available online [https://bsms.ac.uk/helix-of-love].

We are pleased to have shown that arts-based methods can create a space where people feel comfortable to explore different narratives, centre different identities, and challenge assumptions about life with rare conditions. Additionally, we have demonstrated how arts-based approaches can elucidate dimensions of health and illness in ways that augment professional understanding, playing an important role in development and training and policy making.

**References**

- [1] Fraser KD, al Sayah F. Arts-based methods in health research: A systematic review of the literature. *Arts & Health* 2011; 3: 110–145. https://doi.org/10.1080/17533015.2011.561357
- [2] Gorman R, Farsides B. Writing the worlds of genomic medicine: experiences of using participatory-writing to understand life with rare conditions. *Medical Humanities* 2022; 48(.e4) https://doi.org/10.1136/medhum-2021-012346.
- [3] Gorman R, Farsides B, Gammidge T. Stop-motion storytelling: Exploring methods for animating the worlds of rare genetic disease. *Qualitative Research* 2022; 23(6):1737-1758. https://doi.org/10.1177/14687941221110168
- [4] Gorman R, Farsides B, Bonner M. Crafting representations of rare disease: collage as qualitative inquiry. *Arts & Health* 2023; https://doi.org/10.1080/17533015.2023.2254328

## P7

### ‘The painful spot’: psychological support for the couple relationship when parenting children with undiagnosed genetic syndromes (SWAN)

### Amy Hunter^1*^, Holly Baker^1,2^, Kym Winter^3^, Chris Vincent, Joseph Keenan^4^

#### ^1^Genetic Alliance UK, London, UK. ^2^Open University, Milton Keynes, UK. ^3^Rareminds, Hertford, UK. ^4^Manchester Metropolitan University, Manchester, UK

*Corresponding author: amy.hunter@geneticalliance.org.uk

***Orphanet Journal of Rare Diseases*** 2024, 18(1): P7

**Background:** Many children with syndromic conditions remain undiagnosed, despite progress with genomic technology. This is known as SWAN or syndrome without a name. The number of children with an undiagnosed condition born annually is probably in the thousands in the UK alone, and the national support group SWAN UK [https://geneticalliance.org.uk/support-and-information/swan-uk-syndromes-without-a-name/] currently has 4000 families registered for their services.

The complexity and severity of undiagnosed children’s needs can place huge stress on their parents. There is also uncertainty about children’s development or the chances of deterioration or death. Being undiagnosed is isolating because there is no diagnostic label on which parents can base their communication with family, friends and professionals.

A healthy couple relationship is an emotional resource for the parents and the child – without it, families can start to unravel. The health and social care system in place for children in the UK does not offer parents psychological support in their role as parents. Recognising this gap, Genetic Alliance UK [https://geneticalliance.org.uk/] works with the charity Rareminds [https://www.rareminds.org/] to provide couples counselling for parents with an undiagnosed child, free of charge. We find that demand far outstrips supply.

**Methods:** We carried out semi-structured interviews with couples and therapists to explore the relationships of couples using the service, their experiences as parents, and the impact of the counselling service.

**Results:** Our findings draw out themes which highlight the pressure that is placed on couples and the positive impact of the counselling on parents as individuals, on their communication as a couple, and on other dynamics in the family. Therapists reflected on the distinct nature of this client group and the challenges in drawing out the painful but important issues (‘the painful spot’) facing them.

**Conclusions:** The counselling ensures that couples feel heard, understood and respected, and allows them to develop new ways of thinking about difficult couple dynamics and potential coping strategies.

**Acknowledgements**

This work was supported by the Robert Luff Foundation. We would like to thank the parents and therapists who took part in the study.

## P8 ADhoc–An immersive serious game that raises awareness among healthcare professionals about announcing a diagnosis of rare diseases

### Caroline Wernert-Iberg^1,2,6^, Dorothée Leroux^1,6^, Marilyne Oswald^2,6^, Pascal Dureau^3^, Catherine Vignal-Clermont^3,4^, Catherine Vincent-Delorme^5^, Elise Schaefer^6^, Russell Wheeler^7^, Hélène Dollfus^1,2,6^

#### ^1^ERN-EYE Network, Hôpitaux Universitaires de Strasbourg, France. ^2^Filière SENSGENE, Hôpitaux Universitaires de Strasbourg, France. ^3^Hôpital Fondation Adolphe de Rothschild, Paris, France. ^4^Hôpital National des 15-20, Paris, France. ^5^CHRU de Lille, France. ^6^Hôpitaux Universitaires de Strasbourg, France. ^7^ERN-EYE Supporting Partner.

***Orphanet Journal of Rare Diseases*** 2024, 18(1): P8

**Background:** Announcing a diagnosis is an aspect of a doctor's profession that is often delicate and not necessarily part of their training.

To help them improve, The French rare diseases network SENSGENE [www.sensgene.com] has created an innovative and didactic Serious Game [https://prod09.almedia.fr/erneye/] with the aim of raising awareness among healthcare professionals of all ages and specialties about best practices when announcing a diagnosis of rare diseases. The European Reference Network ERN-EYE [www.ern-eye.eu] translated it into English.

The serious game called “ADhoc” aims to remind players of good practice and allow them to immerse themselves in real-life situations. Facing a surprised or disoriented patient, calming a father's anger, or managing unruly siblings, all while delivering a complex diagnosis—these are the objectives drawn from real-life situations that players are confronted with (Figure 1).

**Figure 1:**
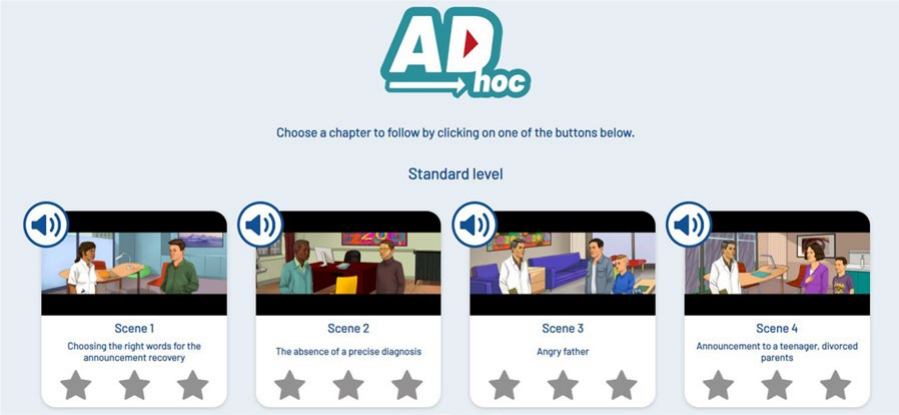
Example of different scenes of the serious game

**Materials and methods:** This serious game was scripted during focus groups by a committee of medical experts from the SENSGENE and ERN-EYE networks. Patient associations and psychologists were also consulted to validate the content.

To make the content fun and original, SENSGENE turned to Almédia (www.almedia.fr) to develop the game and asked Metapraxis [https://metapraxis.fr] to act as assistant project manager.

The Serious Game can be accessed on computers, tablets and smartphones at [https://prod09.almedia.fr/erneye/]

**Results:** ADhoc's main objective is to learn where you want, when you want and quickly! In the role of a doctor in 15 short scenes separated into 3 levels of difficulty (Figure 2), the player enhances their expertise and measures progress through the "best practices" and "empathy" gauges (Figure 3), which fluctuate based on the responses (Figure 4). In addition, at the end of each scene, the player can review the key pedagogical concepts to remember (Figure 5).

**Figure 2:**
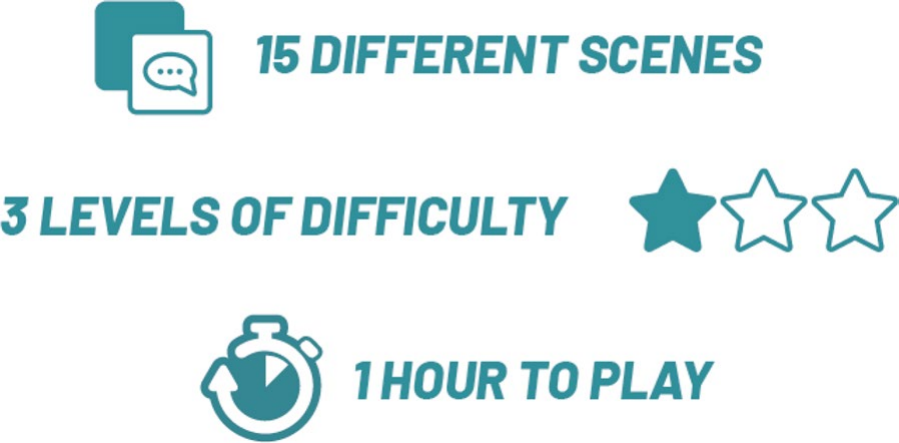
15 short scenes separated into 3 levels of difficulty, 1 hour of gameplay in total

**Figure 3:**
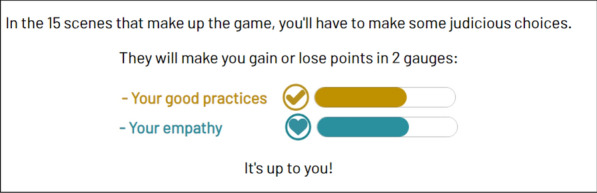
Example of the gauges

**Figure 4:**
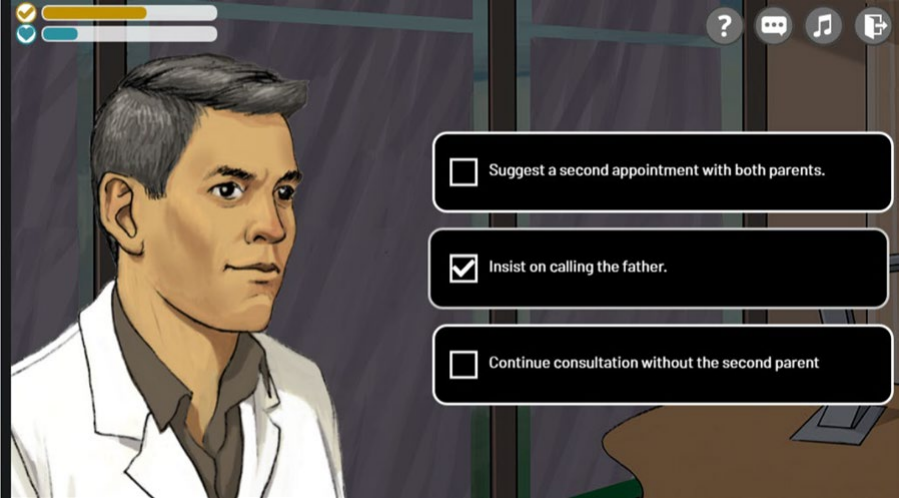
Example of the questions

**Figure 5:**
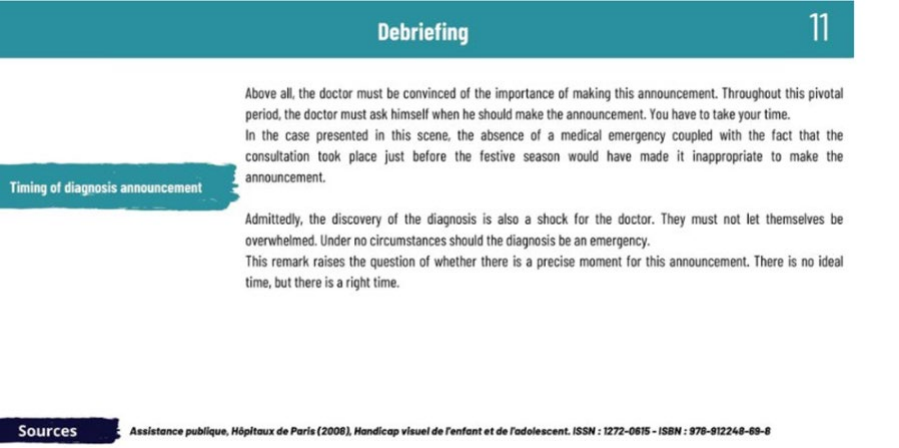
Example of key pedagogical concepts to remember at the end of each scene

**Figure 6:**
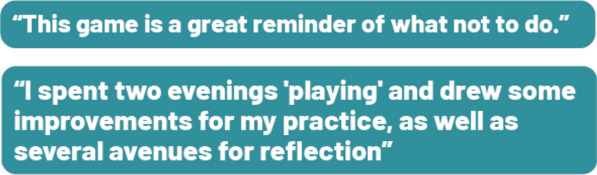
Feedbacks on the serious game

**Conclusion:** Diffused since April 2023, more than 600 players (rare diseases healthcare professionals) logged into the game, with more than a third playing all 15 scenes offered. This Serious Game has been a success since its launch with a rating of 4.8/5 and good feedbacks (Figure 6).

**Acknowledgements**

The Committee of medical experts was composed of Pr Hélène Dollfus, Dr Pascal Dureau, Dr Elise Schaefer, Dr Catherine Vignal-Clermont and Dr Catherine Vincent-Delorme. The serious game was translated and edited with the help of Russell Wheeler, supporting partner of ERN-EYE.

## P9 Introducing PaLaDIn: improving the use of rare NMD patient data to inform healthcare decision making

### Rebecca Leary^1^*, David Allison^2^, Ken Kahtava^3^, Elisa Ferrer^4^, Martijn G Kersloot^5^, Marco Roos^6^, Ilaria Zito^7^, Benedikt Schoser^8^, Bouchra Ezzamouri^9^

#### ^1^John Walton Muscular Dystrophy Research Centre, Newcastle University and Newcastle Hospitals NHS Foundation Trust, Newcastle Upon Tyne, UK. ^2^TREAT-NMD Service Ltd., Newcastle upon Tyne, UK. ^3^FSHD Society, Randolph, USA. ^4^Aparito Espanya, S.L., Barcelona, Spain. ^5^Department of Medical Informatics, Amsterdam UMC location University of Amsterdam and Amsterdam Public Health, Methodology & Digital Health, Amsterdam, The Netherlands. ^6^Department of Human Genetics, Leiden University Medical Center, Leiden, The Netherlands. ^7^Parent Project aps, Rome, Italy. ^8^LMU Klinikum Ludwig-Maximilians-Universität München, Munich, Germany. ^9^Duchenne UK, London, UK

*Corresponding author: becca.leary@newcastle.ac.uk

***Orphanet Journal of Rare Diseases*** 2024, 18(1): P9

The Patient Lifestyle and Disease Data Interactium (PaLaDIn), is an ambitious four-year Innovative Health Initiative (IHI) funded initiative which launched on 1st January 2024. PaLaDIn [https://www.project-paladin.eu/] will drive innovative real world data collection from patients with rare neuromuscular diseases (NMDs) by developing a state-of-the-art, FAIR by design, data collection platform “The Interactium”.

Collecting data from patient registries, Patient Reported Outcome Measures (PROMS), patient preferences, and data from video and wearable devices, the Interactium will provide insights to accelerate novel drug development, improve PROMS and inform health care decision making at all levels. This will be supported by the co-creation of new digital outcome measures using wearable devices and video that meet patient preferences and needs; the development and publication of new datasets for Myotonic Dystrophy (DM) and FSHD; improved access to clinical trials by implementing tools from the Duchenne muscular dystrophy (DMD) and FSHD communities in other diseases.

PaLaDIn will maximise the utility, interoperability and reusability of the Interactium data so it can be widely used by researchers and industry, in clinical trial planning, drug development and understanding of disease. Medicines developers in academia and industry will have access to the data that clearly elicits patients’ preferences and needs, which is particularly important in rare diseases, where outcome measures and patient preference data is scarce. The data from the Interactium will then be used to inform better decision making in healthcare, medicines development and health technology assessments.

Four use cases have been identified to test and improve the Interactium use of rare NMD patient data, these are follows:

1. Use patient data to inform regulatory decision making.
2. Monitor patient care.
3. Create standards of care guidelines based on patient data.
4. Facilitate successful clinical trials.

Learnings from the project will be shared with those working in other rare disease areas via a

series of tools, workshops, and training materials.

**Acknowledgements**

This project is supported by the Innovative Health Initiative Joint Undertaking (IHI JU) under grant agreement No 101132943. The JU receives support from the European Union’s Horizon Europe research and innovation programme, COCIR, EFPIA, EuropaBio, MedTech Europe, Vaccines Europe (and the FSHD Society and TREAT-NMD Services Ltd as contributing partners participating in the project) The project has received funding from UK Research and Innovation (UKRI) under the UK government’s Horizon Europe funding guarantee [grant numbers 10105921, 10103989, and 10083579]

## P10 Building and accessing highly specialized expertise – cross-border healthcare in the European Reference Network for Rare Neurological Diseases

### Tamara Martin^1^, Alisa Jemelka^3^, Carola Reinhard^1^, Holm Graessner^1,2^

#### ^1^Institute of Medical Genetics and Applied Genomics, University of Tübingen, Tübingen, Germany. ^2^Centre for Rare Diseases, University Hospital Tübingen, Tübingen, Germany. ^3^Centre for Rare Diseases, University Hospital Schleswig-Holstein, Lübeck, Germany

***Orphanet Journal of Rare Diseases*** 2024, 18(1): P10

**Background:** Since 2017, the European Reference network for Rare Neurological Diseases (ERN-RND) has been utilizing Multidisciplinary Team (MDT) online consultations for establishing and sustaining cross-border healthcare pathways to shorten and end the diagnostic odyssey, deliver state-of-the-art, targeted and safe therapeutic treatments as well as share knowledge [1].

**Methods:** Supplementary to the IT-platform ‘Clinical Patient Management System’ (CPMS) provided to all ERNs, ERN-RND uses (i) self-commitment of cases and MDT participations, (ii) schedule of regular, fixed appointments in addition to ad hoc meeting organization as well as (iii) two-level organizational structures (local CPMS representatives in healthcare providers; technical support by ERN coordination) as organizational measure to ensure active network-wide use of the MDT consultations.

**Results:** Care pathways focused on ERN-RND-specific highly specialized healthcare services are an instrument to homogenize care and guide the cross-border healthcare activities by providing clear use cases and procedures. They are the result of a structured analytic process focused on care inequalities.

ERN-RND has established 5 specific cross-border healthcare pathways so far (Figure 1):

- Metachromatic leukodystrophy treatment eligibility for gene therapy and stem cell transplantation.
- Deep Brain Stimulation for Dystonia.
- Neuroradiology advice for RND-patients.
- Neurorehabilitation for complex RND-patients.
- Disease-Group specific consultations on diagnosis and disease management.

**Figure 1:**
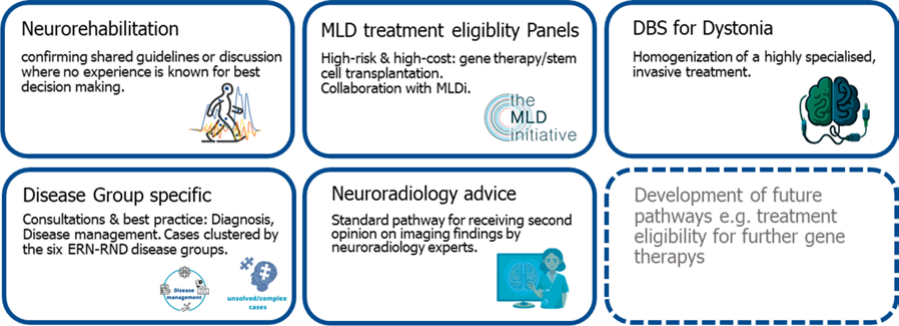
Specific cross-border healthcare pathways in ERN-RND’s approach to cross-border healthcare has provided satisfactory results so far. The network is among the most active users of the CPMS across ERNs. In 2023 alone, 83 patients have been enrolled and discussed in 40 online meetings of international experts. Since its release in 2017, a total of 265 clinicians used ERN-RND CPMS and 299 RND patients have been enrolled [2].

**Conclusions:** In summary, ERN-RND’s experiences show that cross-border healthcare needs clear procedures and care pathways for transfer of cases from national to EU level, stable central & local administration with sufficient funding and dedicated staff in addition to a robust, secure and user-friendly IT platform.

**References**

- [1] ERN-RND. https://www.ern-rnd.eu/. Accessed May 28 2024.
- [2] CPMS KPI tool for ERN coordinators. Active Users and Numbers of panels newly created Nov 2027–Apr 2024 [https://cpms.ern-net.eu/insight/ERN-RND/#/reports-activity]

## P11 Targeting senescence as a novel pharmacological approach in Lymphangioleiomyomatosis

### Clara Bernardelli^1^, Piera Selvaggio^1^, Silvia Rosa^1^, Elisabetta Di Fede^1,2^, Esi Taci^1,2^, Valentina Massa^1,2^, Cristina Gervasini^1,2^, Elena Lesma^1^

#### ^1^Department of Health Sciences, University of Milan, Milan, Italy. 2 “Aldo Ravelli” Center for Neurotechnology and Experimental Brain Therapeutics, Università degli Studi di Milano, Milan, Italy

***Orphanet Journal of Rare Diseases*** 2024, 18(1): P11

**Background:** Lymphangioleiomyomatosis (LAM) is a multisystem ultra-rare disease that affects women[1]. LAM cells, characterized by the constitutive activation of mTOR (mechanistic Target of Rapamycin) due to the impaired expression of its regulatory proteins hamartin or tuberin, invade the lung, leading to the loss of pulmonary function [2]. Rapamycin is the only drug approved for LAM, but new therapies are needed, since LAM relapses to rapamycin discontinuation[3]. mTOR is a driver of senescence[4], a stress-response process characterized by the inhibition of the cell cycle and by the acquisition of a senescence-associated secretory phenotype (SASP) [5]. Through SASP, senescent cells reinforce their phenotype and promote senescence in neighbouring cells[6]. However, dysregulation of senescence might contribute to disease onset, as SASP boost a proinflammatory microenvironment and the cell cycle arrest limits tissue regeneration[7]. We recently demonstrated that primary tuberin-deficient LAM/TSC cells have senescent features depending on mTOR hyperactivation and, in an *in vitro* model of LAM microenvironment, they have the capability to induce senescence in tuberin-expressing primary lung fibroblasts (PLFs) through their conditioned medium (CM), with increased secretion of Interleukin(IL)-8[8]. Interestingly, the lysine acetyl transferase p300 was recently identified as epigenetic driver of cellular senescence and it is known to interact with mTOR[9, 10].

**Materials and methods:** Changing in senescent features, as the increased β-galactosidase positivity and p21 expression, were measured in PLFs grown in the presence of IL-8 and in LAM/TSC cells treated with a monoclonal antibody or with the small molecule SB225002 targeting the IL-8 receptor CXCR2. Then SB225002 was added to PLFs in LAM/TSC CM to counteract senescence induction. In addition, phosphorylation of H2A.X histone and cleaved Caspase 3 were assessed in senescent induced lymphoblastoid cell line (SI-LCLs) derived from controls (HD) and individuals with p300 haploinsufficiency due to germline pathogenetic variants (*EP300+/*).

**Results:** The addition of IL-8 in PLFs CM increased their senescence proportionally to its concentration. Moreover, blocking CXCR2 impaired the capability of LAM/TSC CM to induce senescence in PLFs. At the same time, targeting CXCR2 hampered senescence in LAM/TSC cells. Finally, the impaired phosphorylation of H2A.X in HD SI-LCLs compared to *EP300+/-* LCLs confirmed the role of p300 in senescence, suggesting possible avenues for targeting its activity in LAM.

**Conclusions:** Our results indicate that the modulation of senescence targeting mTOR downstream effectors and non-mTOR regulated mechanisms is an intriguing novel pharmacological approach to interfere with the pathological communication in LAM microenvironment with possible future application in LAM therapy.

**References**

- [1] Johnson SR, Taveira-DaSilva AM, Moss J. Lymphangioleiomyomatosis. Clin Chest Med. 2016 Sep;37(3):389–403.
- [2] McCarthy C, Gupta N, Johnson SR, Yu JJ, McCormack FX. Lymphangioleiomyomatosis: pathogenesis, clinical features, diagnosis, and management. Lancet Respir Med. 2021 Nov;9(11):1313–27.
- [3] Valianou M, Filippidou N, Johnson DL, Vogel P, Zhang EY, Liu X, et al. Rapalog resistance is associated with mesenchymal-type changes in Tsc2-null cells. Sci Rep. 2019 Dec 28;9(1):3015.
- [4] Kennedy BK, Lamming DW. The Mechanistic Target of Rapamycin: The Grand ConducTOR of Metabolism and Aging. Cell Metab. 2016 Jun;23(6):990–1003.
- [5] Hernandez-Segura A, Nehme J, Demaria M. Hallmarks of Cellular Senescence. Vol. 28, Trends in Cell Biology. Elsevier Ltd; 2018. p. 436–53.
- [6] Ortiz-Montero P, Londoño-Vallejo A, Vernot JP. Senescence-associated IL-6 and IL-8 cytokines induce a self- and cross-reinforced senescence/inflammatory milieu strengthening tumorigenic capabilities in the MCF-7 breast cancer cell line. Cell Communication and Signaling. 2017 Dec 4;15(1):17.
- [7] Coppé JP, Desprez PY, Krtolica A, Campisi J. The Senescence-Associated Secretory Phenotype: The Dark Side of Tumor Suppression. Annual Review of Pathology: Mechanisms of Disease. 2010 Jan 1;5(1):99–118.
- [8] Bernardelli C, Ancona S, Lazzari M, Lettieri A, Selvaggio P, Massa V, et al. LAM Cells as Potential Drivers of Senescence in Lymphangioleiomyomatosis Microenvironment. Int J Mol Sci. 2022 Jul 1;23(13).
- [9] Son SM, Park SJ, Breusegem SY, Larrieu D, Rubinsztein DC. p300 nucleocytoplasmic shuttling underlies mTORC1 hyperactivation in Hutchinson-Gilford progeria syndrome. Nat Cell Biol. 2024 Feb;26(2):235–49.
- [10] Sen P, Lan Y, Li CY, Sidoli S, Donahue G, Dou Z, et al. Histone Acetyltransferase p300 Induces De Novo Super-Enhancers to Drive Cellular Senescence. Mol Cell. 2019 Feb 21;73(4):684-698.e8.

## P12 Bridging global disparities: an analytics pipeline for detecting *bias* and incompleteness in rare diseases datasets

### Alenka Guček^1,2,^*, Matej Kovačič^1^, Tanja Zdolšek Draksler^1,2^

#### ^1^Department for Artificial intelligence, Jožef Stefan Institute, Ljubljana, Slovenia. ^2^IDefine Europe, Slovenia

*Corresponding author: alenka.gucek@ijs.si

***Orphanet Journal of Rare Diseases*** 2024, 18(1): P12

In rare diseases, limited scientific and clinical data often consist of single case reports and local data with small populations. To unravel these complexities, global cohorts' data is crucial, making patient-reported outcomes (PROs) and caregiver-reported outcomes (CROs) repositories essential for insights. However, bridging global disparities poses challenges, as detecting bias and data incompleteness in these repositories is pronounced, with notable disparities in data representation across continents. While a comprehensive understanding requires global data, the reality is that data predominantly comes from Western countries with more developed healthcare systems. This imbalance led our research team to develop an advanced analytics pipeline explicitly designed to quantify and address the incompleteness of data originating from regions with less developed healthcare systems.

Our analytics pipeline (Fig 1) integrates sophisticated statistical and AI methodologies to systematically evaluate and eliminate or try to correct the bias inherent in PROs and CROs repositories. By automating the identification and mitigation of data incompleteness, the pipeline ensures a more nuanced and representative depiction of the global rare disease landscape. Notably, the pipeline contributes to minimizing the impact of geographic disparities in rare disease data, promoting a more equitable foundation for research and healthcare advancements. In the pilot version, the pipeline is focused on the Kleefstra syndrome CROs data from the Genida registry.

**Figure 1:**
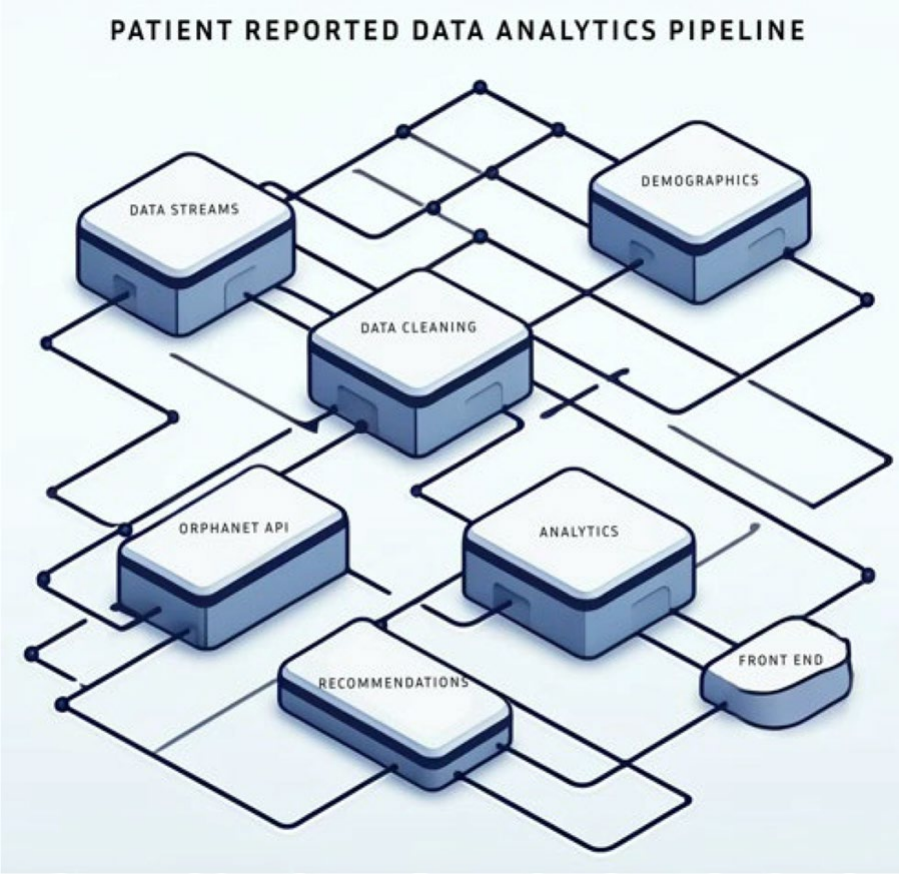
Architecture of analytics pipeline that measures data incompleteness (and hence bias in data) of PRO& CRO in rare disease data registries

Moreover, the pipeline's capabilities not only enhance data reliability but also shed light on the unique challenges faced by underrepresented groups and regions. By quantifying and addressing the imbalances in data, our research aims to facilitate a more inclusive and equitable approach to rare disease research, ultimately leading to more effective interventions and improved outcomes on a global scale.

**Acknowledgements**

This research was supported by grant from the European Union (AI4Gov - 101094905). We thank GenIDA for providing the data.

## P13 Mutual knowledge exchange (MKE): a cost-effective research methodology for knowledge generation of patient-oriented knowledge in rare diseases

### Gry Velvin*, Heidi Olsson, Taran Youssefian Blakstvedt

#### TRS, National Resource Centre for Rare Disorders, Research and Innovation Department, Sunnaas Rehabilitation Hospital

*Corresponding author: Gry.velvin@sunnaas.no

***Orphanet Journal of Rare Diseases*** 2024, 18(1): P13

**Background:** The mandate of TRS National Resource Centre for Rare Disorders is to generate and disseminate practice-related knowledge about rare diagnoses. After conducting comprehensive systematic searches in relevant databases for pertinent literature and research, we identified a significant gap in existing knowledge, particularly concerning the psychosocial aspects and physical functioning of individuals with Stickler syndrome. This discovery is especially notable given the abundance of comprehensive medical research in related fields, such as audiology, ophthalmology, genetics, and diagnostic.

**Methodology for knowledge generation:** In response to the identified knowledge gap and in accordance with our mandate to gather, generate and disseminate knowledge, we initiated a course for families diagnosed with Stickler syndrome, framing it as a reciprocal learning retreat, combined with a research project.

The primary purpose was twofold:

1. To enhance the health literacy of participants diagnosed with Stickler and their relatives.
2. To enable professionals to learn from the experiences of these patients and increase the knowledge base on the rare disease.

The course spanned three days and included a series of expert presentations and informational sessions for improving health literacy for the participants. Concurrently, the participants completed a questionnaire addressing demographic, medical, and psychosocial factors, including standardized validated instruments measuring pain, fatigue, quality of life and daily function. Additionally, they engaged in semi-structured focus group interviews to gather in-depth knowledge about the same issues emphasized in the quantitative part of the study.

**Methodological utility:** This method proved to be resource efficient, targeted, and provided unique insight in an area lacking knowledge. For all the participants in the MKE, the expenses were covered for living, travel, and lost earnings. Moreover, the participants were highly motivated to share their experiences and insights of living with the disease. For the researchers, this approach facilitated easy access to motivated participants willing to contribute. The MKE-methodology also enabled the researchers to acquire unique new knowledge rapidly and efficiently in an uncharted area, which is crucial for knowledge dissemination. Knowledge that is important for understanding the needs of individuals with this rare disease. In addition, the patients reported that the knowledge gained through participating in the program was valuable. They emphasized the significance of meeting other patients in the same situation and sharing their own experiences in a broader context highlighted as research. From a societal perspective, this represents a highly cost-effective methodology that is economically viable and swiftly garners unique knowledge.

## P14 Belgian national action plan: financing and monitoring Medical Centers of Human Genetics’ participation to EQAs focused on rare diseases

### Joséphine Lantoine^1^*, Valérie Benoit^2^, Anne Brysse^3^, Kathleen Claes^4^ , Anniek Corveleyn^5^ , Vinciane Dideberg^3^, Martine De Rycke^6^ , Lut Van Laer^7^ , Xavier Peyrassol^8^ , Magali Philippeau^9^ , Marie Ravoet^9^, Sonia Rombout^2^, Sara Seneca^6^, Sofie Symoens^4^ , Kris Van den Bogaert^5^, Françoise Wilkin^8^, Wim Wuyts^7^, Arnaud Capron^1^

#### ^1^Department of Quality of Laboratories, Sciensano, rue Juliette Wystman 14, 1050 Ixelles, Belgium. ^2^Institut de Pathologie et de Génétique, Avenue Georges Lemaître 25, 6041 Gosselies, Belgium. ^3^Centre de Génétique Humaine (UNILAB) - CHU Sart-Tilman, Domaine Universitaire du Sart Tilman. (Bâtiment Tour 6, +1), B-4000 Liège, Belgium. ^4^Centrum Medische Genetica - UZ Gent, Corneel Heymanslaan 10, 9000 Gent, Belgium. ^5^Centrum Menselijke Erfelijkheid - UZ Leuven, Herestraat 49, 3000 Leuven, Belgium. ^6^Centrum voor Medische Genetica - UZ Brussel Vrije Universiteit Brussel, Laarbeeklaan 101, 1090 Brussels, Belgium. ^7^Centrum Medische Genetica - UZ Antwerpen, Prins Boudewijnlaan 43 Bus 6, 2650 Edegem, Belgium. ^8^Centre de Génétique Humaine – ULB Erasme, Route de Lennik 808, 1070 Anderlecht, Belgium. ^9^Centre de Génétique Humaine - Cliniques Universitaires Saint-Luc UCL, Avenue Hippocrate 10, 1200 Brussels, Belgium

*Corresponding author: josephine.lantoine@sciensano.be

***Orphanet Journal of Rare Diseases*** 2024, 18(1): P14

**Aims:** In order to support the Belgian Medical Centers of Human Genetics (BMCHGs) in the development of a Quality Management System and participation to External Quality Assessment programs (EQAs), Belgian healthcare authorities have developed a funding through a national action^1^ plan. One of the funded actions concerns the BMCHGs’ participation to EQAs focused on genetic tests performed in the context of hereditary rare diseases and cancers.

**Method:** A working group composed of representatives of the eight BMCHGs and one quality officer from the Belgian Institute for health, Sciensano was established in 2019. It is in charge of the yearly establishment of the financeable EQAs’ list based on (i) the offer from EQA providers and (ii) clinical relevance of the EQA. The working group also published guidelines regulating the participation to EQAs in 2021^2^.

**Results:** In 2023, the working group has selected 109 financeable EQAs. In total, Belgian healthcare authorities have financed 155 EQAs to the BMCHGs representing a financing of 87.169,75 euros.

Sciensano monitored the performances of the BMCHGs to those EQAs. We list only 5 poor performance among the 136 financed EQAs for which we already received a performance report. For 70 of them, the centers have obtained a satisfactory performance score in all categories of assessment (genotyping/analysis; interpretation and clerical accuracy). The interpretation is the category of assessment where BMCHGs have mostly done errors (41 EQA /136) but without impact on the global satisfactory score.

**Conclusion:** The BMCHGs have performed well in 2023 with only few poor performances. Nevertheless, it is important to continue to participate to those quality controls for the sake of continual improvement. We saw that the financing granted to the BMCHGs have allowed them to increase their participation to quality controls thereby supporting them in their willingness of improvement.

**References**

- [1] Onkelinx L, Plan belge pour les Maladies Rares. Ministère des Affaires Sociales et de la Santé Publique, Bruxelles, Décembre 2013
- [2] Lantoine J, Brysse A, Dideberg V, Claes K, Symoens S, Coucke W, et al..; Frequency of Participation in External Quality Assessment Programs Focused on Rare Diseases: Belgian Guidelines for Human Genetics Centers. JMIR Med Inform. 2021 July 12;9(7):e27980 Available from: https://medinform.jmir.org/2021/7/e27980

## P15 Tackling the invisibility of RD in European Member States: the OD4RD project contribution

### Sylvie Maiella^1^, Marie-Cécile Gaillard^1^, Kurt Kirch^2^, Carina Thomas^2^, Marta Fructuoso^1^, Caterina Lucano^1^, Elsa Ekblom^3^, Charlotte Rodwell^1^, Valérie Serrière-Lanneau^1^, Julie Tarahoui^1^ , Marc Hanauer^1^, Stefanie Weber^2^, Rula Zain^3^ & Ana Rath^1^

#### ^1^US14, Inserm, Paris, France. ^2^Federal Institute for Drugs and Medical Devices (BfArM), Germany. ^3^Centre for Rare Diseases, Department of Clinical Genetics, Karolinska University Hospital, Stockholm, Sweden

***Orphanet Journal of Rare Diseases*** 2024, 18(1): P15

The OD4RD/OD4RD2 projects tackle the invisibility of rare diseases (RD) in European member states’ health systems, promotes harmonisation of practice and facilitate generation of standardised interoperable data around RD, thus contributing to meet the RARE 2030 ambitions concerning data.

The project supports the production of the ORPHAcodes, the only terminology that makes visible all 6000+ RD in Health Information Systems (HIS), recognised as a Best practice by the European Commission [1] as the exploitation of ORPHAcodes’ annotated Health data increases the visibility of people living with a RD [2] [3] [4] [5] and contributes to ensure that needs of all RD Patients are met. ORPHAcodes update is carried out in collaboration with European Reference Networks (ERNs) to fit coders’ needs [6]. ORPHAcodes are made available with annotations, definitions and transcoding information to facilitate the coding work and reduce its burden. Finally, they are delivered in different formats, adaptable to the different settings [7].

The project provides coordinated support for ORPHAcodes implementation in hospitals hosting ERNs in 20 countries, thanks to the Network of Orphanet National Nomenclature Hubs. The Hubs make available a National Helpdesk and organise trainings and events around ORPHAcoding to build capacity so that information is collected correctly and uniformly. The Network of Hubs allows to mutualise the success stories and address common problems.

A survey to evaluate ORPHAcodes usage by Health Care Providers linked to ERNs has been carried out. Each Hub contacted national ERN units as per registration in the Orphanet Database [8] and submitted a common minimum subset of 7 questions.

The preliminary results, deriving from the analysis of 12 survey reports on 440 ERN units across EU, indicate that approximately 53% of them use ORPHAcodes, while main reasons for not using ORPHAcodes are, in decreasing importance order: Unavailability in Electronic Health Record, Use not mandatory, Lack of time/resources, Codes not suitable and Lack of knowledge.

The survey results will be complemented with the JARDIN Joint Action survey results, to obtain a complete picture of practices by ERN and across countries and identify barriers and needs. Results will be discussed with the ERNs during a dedicated day to raise awareness about ORPHAcoding benefits and to discuss around ERN data strategy so as to refine the project’s national and transversal actions to improve harmonisation.

**Acknowledgements**

We would like to thank all the Orphanet Nomenclature National Hubs teams [https://od4rd.eu/02-partners ] who participate in the project and contributed their data for the analysis here presented. The OD4RD & OD4RD2 projects are Co-Funded by the European Union. Views and opinions expressed are however those of the authors only and do not necessarily reflect those of the European Union or HADEA. Neither the European Union nor the granting authority can be held responsible for them.

**References**

- [1] [https://health.ec.europa.eu/european-reference-networks/rare-diseases_en]
- [2] ORPHAcodes use for the coding of rare diseases: comparison of the accuracy and cross country comparability. Mazzucato M, Pozza LVD, Facchin P, Angin C, Agius F, Cavero-Carbonell C, Corrochano V, Hanusova K, Kirch K, Lambert D, Lucano C, Maiella S, Panzaru M, Rusu C, Weber S, Zurriaga O, Zvolsky M, Rath A. Orphanet J Rare Dis. 2023 Sep 4;18(1):267. https://doi.org/10.1186/s13023-023-02864-6. PMID: 37667299
- [3] A retrospective review of the contribution of rare diseases to paediatric mortality in Ireland. Gunne E, McGarvey C, Hamilton K, Treacy E, Lambert DM, Lynch SA. Orphanet J Rare Dis. 2020 Nov 4;15(1):311. https://doi.org/10.1186/s13023-020-01574-7. PMID: 33148291 Free PMC article.
- [4] Healthcare burden of rare diseases in Hong Kong – adopting ORPHAcodes in ICD-10 based healthcare administrative datasets. Chiu ATG, Chung CCY, Wong WHS, Lee SL, Chung BHY. Orphanet J Rare Dis. 2018 Aug 28;13(1):147. https://doi.org/10.1186/s13023-018-0892-5. PMID: 30153866 Free PMC article.
- [5] The interoperability between the Spanish version of the International Classification of Diseases and ORPHAcodes: towards better identification of rare diseases. Rico J, Echevarría-González de Garibay LJ, García-López M, Guardiola-Vilarroig S, Maceda-Roldán LA, Zurriaga Ó, Cavero-Carbonell C. Orphanet J Rare Dis. 2021 Mar 9;16(1):121. https://doi.org/10.1186/s13023-021-01763-y. PMID: 33750434
- [6] [https://github.com/OD4RD/Main-Help-Desk/wiki/8.-Orphanet%E2%80%90ERN-collaborations]
- [7] [https://www.orphadata.com/orphanet-nomenclature-for-coding/]
- [8] [https://www.orpha.net/en/expert_centres/european-reference-network]

## P16 Mobility assessment for individuals with arthrogryposis multiplex congenita: content validation of mobility measures based on the international classification of functioning, disability, and health

### Ahlam Zidan^1*^, Jaclyn Sions^2^, Alexa Cirillo^3^, Verity Pacey^4^, Laurie Snider^1^, Kristen Donlevie^5^, Noémi Dahan-Oliel^1,3^

#### ^1^Faculty of Medicine and Health Sciences, McGill University, Montreal, Quebec, Canada. ^2^Department of Physical Therapy, University of Delaware, Newark, Delaware, USA. ^3^Shriners Hospitals for Children, Montreal, Quebec, Canada. ^4^Department of Health Sciences, Macquarie University, Sydney, Australia. ^5^Department of Occupational Therapy, Boston University, Boston, USA.

*Corresponding author: ahlam.zidan@mail.mcgill.ca

***Orphanet Journal of Rare Diseases*** 2024, 18(1): P16

**Background:** Arthrogryposis multiplex congenita (AMC) is a spectrum of non-progressive congenital conditions characterized by multiple congenital contractures [1,2]. For AMC mobility assessment, clinicians and researchers employ measures not formally validated for the AMC population [3]. Validated measures can underpin the reliable interpretation of mobility status and the judicious allocation of resources in line with patient needs [4]. This study aimed to estimate the degree to which the mobility dimensions of children with AMC are encapsulated within four commonly used mobility measures following the International Classification of Functioning, Disability, and Health (ICF) framework.

**Materials and methods:** The Functional Mobility Scale (FMS), Gillette Functional Assessment Questionnaire walking scale (FAQ-W), and the mobility domains of the Functional Independence Measure for Children (WeeFIM) and Patient-Reported Outcomes Measurement Information System (PROMIS) were assessed for content validation by two independent raters based on the Cosmin guidance and the refined ICF linking rules. A panel of five experts, including researchers and clinicians with academic and lived experience in AMC, assessed the measures’ content validity by responding to a questionnaire that asked about each item’s comprehensibility, relevance, and comprehensiveness, as well as whether they agreed or not on the meaningful concept linked to the ICF by the two independent raters. The Kappa coefficient was calculated to establish the inter-rater reliability between the two raters [5].

**Results:** The Kappa coefficient of the inter-rater reliability was 0.79 [95% CI: 0.78- 0.84]. The identified meaningful concepts were linked to thirty-four ICF categories. The four mobility measures demonstrated acceptable content validity (Scale-Content Validity Index ranged from 0.82 to 1). The items of the four measures were mainly linked to the activity and participation (mostly the mobility chapter) and the environmental factors domains of the ICF (representing support and assistive devices needed for mobility). Some important concepts for the AMC population were not covered well by any of the four measures, such as scooting, which is more common than crawling in this population, and the type and influence of assistive devices (e.g., motorized or manual wheelchairs) on mobility status.

**Conclusions:** The content of the four measures proved to be valid for children with AMC. The need for support and the use of assistive devices are important concepts when measuring mobility in children with AMC, and the available ICF codes do not comprehensively address these concepts. Findings can help clinicians and researchers select the most relevant outcome measure based on their patients’ needs (Fig 1).

**Figure 1:**
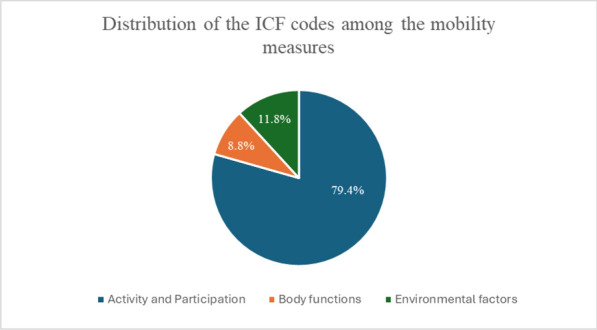
Distribution of the ICF categories among the four mobility measures

**References**

- [1] Dahan-Oliel N, Cachecho S, Barnes D, Bedard T, Davison AM, Dieterich K, Donohoe M, Fąfara A, Hamdy R, Hjartarson HT, S Hoffman N, Kimber E, Komolkin I, Lester R, Pontén E, van Bosse HJP, & Hall JG. International multidisciplinary collaboration toward an annotated definition of arthrogryposis multiplex congenita. American journal of medical genetics. Part C, Seminars in medical genetics. 2019; 181(3): 288–299.
- [2] Ferguson J, Wainwright A. Arthrogryposis. Orthopaedics and Trauma. 2013; 27(3): 171-80.
- Graham HK, Harvey A, Rodda J, Nattrass GR, Pirpiris M. The functional mobility scale (FMS). Journal of Pediatric Orthopaedics. 2004; 24(5):514-20.
- [3] Polit DF, Beck CT. The content validity index: are you sure you know what's being reported? Critique and recommendations. Research in Nursing & Health. 2006; 29(5):489-97.
- [4] Dumuids-Vernet MV, Provasi J, Anderson DI, Barbu-Roth M. Effects of early motor interventions on gross motor and locomotor development for infants at-risk of motor delay: a systematic review. *Frontiers in Pediatrics*. 2022; 10: 877345.

## P17 National Plan/Strategy (NP/NS) for rare diseases in Georgia; involvement of PLWRD

### Oleg Kvlividze ^1,2^, Zhana Chokheli ^1,3^, and the WG on NP/NS for RD in Georgia

#### ^1^Georgian Foundation for Genetic and Rare Diseases (GeRaD), Tbilisi, Georgia, ^2^School of Medicine, New Vision University, Tbilisi, Georgia, ^3^SCN2A-Georgia, Tbilisi, Georgia

***Orphanet Journal of Rare Diseases*** 2024, 18(1): P17

**Background:** In recent years, rare diseases in Georgia as a global socio-clinical problem have experienced a persistent tendency to increase the attention and support from the State, professional and civil society. On June 17, 2022, by order of the Minister of Health (MOH), a working group (WG) was established to create a 2024-2030 National Plan/Strategy (NP/NS) for Rare Diseases (RD).

**Materials and Methods:** When drawing up the NP/NS, the WG, based on EUROCERD recommendations and best experience of EU countries, adhered to the following criteria/topics that should have been reflected in the document: 1.Methodology and Governance of the NP/NS; 2. Definition, Codification and Inventory of RD; 3. Research on RD; 4. Education Issues on RD; 5. Care - Centers of Expertise / European ReferenceNetworks / Cross Border Health Care; 6.Orphan Drugs; 7. Diagnostics and Treatment; 8. Social Services for RD. The NP/NS of Bulgaria, Croatia, Czechia, Romania, Slovakia, and Slovenia were analyzed.

**Results:** Representatives of people living with RD (PLWRD) made up almost 30% of the total WG members. The draft document submitted to the MOH has received approval in November 2023. The official announcement of the NP/NS was presented at an event organized by the GeRaD and dedicated to RDD on February 29, 2024. The final version of the document was agreed with the most of the stakeholders will be approved by the MOH in May 2024. The main vision of the National Strategy/Plan, its goals, as well as 7 objectives were formulated, including issues of management, treatment, education, orphan drugs accessibility, research, and social services related with RD. Patient interests are included broadly in all activities, particularly, the document declares expansion of patients’ role in the decision-making process related to RD policy. The document also spells out mechanisms for monitoring and evaluation the effectiveness of the NP/NS.

**Conclusion:** We hope that NP/NS implementation with an active patients participation will help PLWRD in Georgia to improve their quality of life and reduce the burden of disease for their parents and caregivers.

**Acknowledgements**

The authors express their gratitude to all members of the WG for their great efforts and effective cooperation in creation of the first Georgian NP/NS on RD.

## P18 RDK^TM^: Reducing the rare disease diagnostic odyssey by leveraging the power of the Orphanet rare disease knowledge base

### Charlotte Rodwell^1^*, Valérie Lanneau^1^, Marc Hanauer^1^, Caterina Lucano^1^, Carolina Fabrizzi^1^, Gersende Gendre^1^, Florence Sauvage^1^, Elise de Beauvais^2^, Margaux Sarfati^2^ , Tu Nguyen^2^, David Ker^2^, Robin Sarfati^2^, Bruno Sarfati^2^, Ana Rath^1^

#### ^1^INSERM, US14 – Orphanet, Plateforme Maladies Rares, 96 rue Didot, 75014, Paris, France. ^2^Tekkare/As We Know – 13 rue de la Vanne, campus Soparq, 92120 Montrouge, France

*Corresponding author: charlotte.rodwell@inserm.fr

***Orphanet Journal of Rare Diseases*** 2024, 18(1): P18

**Background:** One of the main challenges faced by rare disease (RD) patients and their families is the diagnostic odyssey, with an average delay-to-diagnosis of 5 years from first symptoms. With around 300 million people living with a rare disease worldwide, this represents a real public health challenge. To address this, Orphanet (INSERM) the RD knowledge base, has partnered with As We Know and Tekkare (specialists in digital and data science) in order to co-develop, through an innovative public-private partnership, a free medical device designed for healthcare providers that aims to reduce diagnostic delay.

**Materials and methods:** Orphanet provides Tekkare with scientific content (nomenclature, epidemiological data, natural history, abstracts, guidelines, classifications), alignments with other terminologies, phenotypic descriptions and genetic data according to the evolution of scientific knowledge and users’ feedback collected in the RDK ^TM^ [1] application. In particular, in order to run the Assistant algorithm, Orphanet provides phenotypic annotations of each RD using Human Phenotype Ontology (HPO) terms, their frequency in a disease, their diagnostic value (diagnostic criterion/pathognomonic sign) when relevant, and the links to expert centres. Tekkare, leveraging its technology expertise, has developed RDK ^TM^ into an innovative, UX-driven mobile and web application providing professionals with all relevant information on over 6300 RDs right in their pockets. Developing features such as the Assistant Tool, expert centre geolocation, enhanced search, and an intuitive user interface.

**Results:** Launched in France in June 2023, RDK^TM^ allows healthcare professionals to identify one or more potential RD and access up-to-date knowledge and information on appropriate expert centres. Users can search for a RD or centre directly or can use the assistant tool. Using the assistant tool, a healthcare professional enters the clinical signs/symptoms observed and patient’s age category to receive a list of diseases associated with these sign/symptoms: the algorithm sorts the results are sorted in order of pertinence. The professional is provided with more information about each RD and the centres for referral to establish a diagnosis.

**Conclusion:** To date RDK^TM^ (desktop and mobile versions) has been visited around 44k times since its launch, and the mobile apps have been downloaded around 3.4k times. This device is available in France (in French and English) and will soon be expanded to other countries and languages. Feedback from end users is sought and integrated to improve the device and the public sources of data, Orphanet and HPO, feeding a virtuous circle for the RD community.

**References**

- [1] Rare Disease Knowledge: RDK^TM^, https://rdk.asweknow.com/en/assistant

## P19 Reference Centers for Rare Diseases in Portugal: How Collaborative Discussions Between Key Stakeholders Contribute to Improve Healthcare for People Living with Rare Diseases

### Raquel Marques^1^, Catarina Costa Duarte^1^, Vanessa Ferreira^2^, Patrícia Lamúrias^3^, Flôr Ferreira^4^, Diana Nunes^4^, Rita Francisco^5^, Paulo Gonçalves^1^

#### ^1^RD-Portugal–União das Associações das Doenças Raras de Portugal, Lisboa, Portugal (https://raras.pt/). ^2^Humanized Solutions, Lisboa, Portugal (https://humanizedsolutions.com/). ^3^Journalist - Associação Atípicas. ^4^ Cognipharma, Lisboa, Portugal (https://www.cognipharma.com/). ^5^EURORDIS – Rare Diseases Europe (https://www.eurordis.org/)

***Orphanet Journal of Rare Diseases*** 2024, 18(1): P19

The principle of creating Reference Centers (RCs) follows a decision by the European Commission to create 24 European Networks ^1^ that began in 2017, with the aim of bringing together and sharing the best knowledge and practices at European level, linking the best working for people with rare, complex and low-prevalence diseases. ^2^

The expertise RCs integrated into these networks are highly specialized institutions that play a crucial role in the treatment of Rare Diseases. In Portugal, Ministerial Order no. 194/2014, of September 30, established the process for identifying, approving and recognizing National RCs for providing health care, namely for the diagnosis and treatment of rare diseases, defining a RC as any service, department or health unit that stands out as the highest exponent of skills in the provision of high-quality healthcare. These centers must include experienced and highly qualified multidisciplinary teams in their field, as well as highly specialized medical structures and equipment.

**RD-Portugal - União das Associações das Doenças Raras de Portugal** is a non-profit organization, in the form of a Federation, which brings together various patient associations. One of its goals is to actively participate in scientific developments in the healthcare of families living with Rare Diseases in Portugal. RD-Portugal is part of the Intersectoral Working Group for Rare Diseases, with the aim of drawing up the National Intersectoral Plan for Rare Diseases. ^3^

In November 2023, the non-profit organized an event under the theme "Reference Centers for Rare Diseases in Portugal and their close relationship with European Reference Networks" with the aim of bringing together various experts (patients, clinicians, researchers, decision-makers, consultants, journalists), invited for their relevant intervention in the areas of Health and/or Rare Diseases, to debate the existing model of healthcare for people living with rare diseases and draw up a final Report, with key-topic conclusions to improve the patient journey, as well as the internal efficiency and effectiveness of the centers. The final Report was shared with all participants, presented publicly, and made available to policymakers.

Through collaborative work with key stakeholders, including KOLs, patients, clinicians, researchers, decision-makers, **RD-Portugal** was able to lead a process that fostered constructive discussion between the different players on the field of rare diseases, developing a Report that translates into an important resource to improve healthcare for people living with rare diseases in Portugal.

## P20 Belgian-Ukrainian cooperation for the identification of some specific needs related to the management of rare diseases in Ukraine and the discussion of improvement measures

### Svitlana Iasechko^1,2^*, Nataliia Gorovenko^3^, Nataliia Samonenko^4^, Nataliia Olkhovych^4^, Elena Kokhanovskaya^5^, Ievgen Miruchin^6^, Dmytro Mikitenko^7^, Anastasiya Babintseva^8^, Iryna Lastivka^8^ , Vitaliy Matyushenko^9^, Olena Kharytonova^10^, Yuliia Hodovanets^8^, Iryna Senyuta^11^, Nataliia Shyshka^2^, Nathalie Monique Vandevelde^1^

#### ^1^Service Quality of Laboratories, Sciensano, Brussels, Belgium. ^2^Kharkiv National University of Internal Affairs, Kharkiv, Ukraine. ^3^National University of Health Care of Ukraine named P.L. Shupyk, Kyiv, Ukraine. ^4^National Children's Specialized Hospital "Okhmatdyt", Kyiv, Ukraine. ^5^Taras Shevchenko National University of Kyiv, Kyiv, Ukraine. ^6^Kharkiv National University V. N. Karazin, Kharkiv, Ukraine. ^7^Lviv Medical Center “Echomed”, Lviv, Ukraine. ^8^Bukovinian State Medical University, Chernivtsi, Ukraine. ^9^Kharkiv charitable foundation “Children with spinal muscular atrophy”, Kharkiv, Ukraine. ^10^National University “Odesa Law Academy”, Odesa, Ukraine. ^11^Danylo Halytsky Lviv National Medical University, Lviv, Ukraine

*Corresponding author: svitlana.iasechko@sciensano.be

***Orphanet Journal of Rare Diseases*** 2024, 18(1): P20

**Background:** The Ukrainian plan for rare diseases (RD) 2021-2026 [1] covers priority actions aiming to improve the (i) patients’ access to specialized diagnosis, medical care, therapy, nutrition, and social-psychological support, (ii) RD’s epidemiological follow-up, (iii) healthcare professionals’ training, (iv) public awareness about RD challenges and prevention, and (v) international scientific multidisciplinary cooperation. In October 2013, a cooperation has been established between the Belgian Institute for Public Health (Sciensano) and Ukrainian scientific experts in order to provide some guidance and support based on the experience gained during the Belgian National RD Plan (published in 2013) [2] , and set-up of Belgian reference medical laboratories of clinical pathology for RD [3].

**Materials and methods:** A working group composed of 7 Ukrainian medical experts, 7 Ukrainian legal experts, and the coordinator of the Belgian reference laboratories for RD has been set-up in December 2023. During the meetings, Ukrainian physicians report specific needs in the medical practice and laboratory medicine requiring new measures or modifications of the Ukrainian legislation. Then, Sciensano compares the Belgian and Ukrainian situation for the reported questions and problems. On that basis, Ukrainian legal experts work on a reasoned legal answer based on a comparison between the Belgian and EU legislation.

**Results:** The Belgian-Ukrainian cooperation has highlighted shortcomings in (i) general practitioners’ education on RD symptomatology and follow-up, (ii) access to traineeships abroad for medical specialists, (iii) parents’ observance to medical consultations for neonates after a positive RD diagnosis during newborn screening, with possible severe consequences for the child related to the delay in access to medical care (e.g. delay in access to nutritional supplements for patients with phenylketonuria), (iv) access to laboratory proficiency testing since the Russian aggression against Ukraine in February 2022. Besides, differences exist between the Ukrainian and EU legislation concerning reference medical laboratories’ recognition and pediatric patients’ rights for access to medical care. Based on the information gathered, the working group develops proposals for amendments to regulatory acts, including the Civil Code of Ukraine [4], in the following areas: parents or guardians’ informed consent on children’s behalf and responsibility in case of refusal to treatment to neonates and children; legal regulation of databases; use of artificial intelligence for RD diagnosis and treatment.

**Conclusions:** This project reflects the Ukrainian willingness to integrate European standards for RD management and healthcare legislation. It strengthens the actions of the Ukrainian RD action plan and highlights the benefits of international collaborations.

**References**

- [1] On approval of the action plan for the implementation of the Concept for the Development of the Medical Care System for Patients Suffering from Rare (Orphan) Diseases for 2021-2026: Ord. of the Cabinet of Ministers of Ukraine. of Ministers of Ukraine dated 11.10.2021 No. 1235-p. URL: https://zakon.rada.gov.ua/laws/show/1235-2021-p#Text
- [2] Plan Belge Maladies Rares. sciensano.be. URL: https://www.sciensano.be/fr/biblio/plan-belge-maladies-rares
- [3] Vandevelde NM, Vermeersch P, Devreese KMJ, Vincent MF, Gulbis B, Eyskens F, Boemer F, Gothot A, Van Hoof VO, Bonroy C, Stepman H, Martens GA, Bossuyt X, Roosens L, Smet J, Laeremans H, Weets I, Minon JM, Vernelen K, Coucke W; Advisory Board of the Action 1 of the Belgian National Plan for Rare Diseases. Belgian rare diseases plan in clinical pathology: identification of key biochemical diagnostic tests and establishment of reference laboratories and financing conditions. Orphanet J Rare Dis. 2021 Feb 17;16(1):89.
- [4] Civil Code of Ukraine: Code of Ukraine dated 16.01.2003 No. 435-IV: as of 27 April. 2024 URL: https://zakon.rada.gov.ua/laws/show/435-15#Text

## P21 DetERminants and impact of diagnostic delay in people living with a RD in Spain

### Juan Benito-Lozano^1^, Greta Arias-Merino^1^, Beatriz Arconada López^2^, Mario Gómez Martínez^1^, Begoña Ruiz García^3^ & Verónica Alonso Ferreira*^1^

#### ^1^Institute of Rare Diseases Research (IIER), Instituto de Salud Carlos III (ISCIII), Madrid, Spain. ^2^Spanish Federation of Rare Diseases (FEDER), Madrid, Spain. ^3^National Reference Centre for care of People with Rare Diseases and their Families (Creer), belonging to Institute for Older Persons and Social Services (Imserso) Burgos, Spain

*Corresponding author: valonso@isciii.es

***Orphanet Journal of Rare Diseases*** 2024, 18(1): P21

**Background:** According to the International Rare Diseases Research Consortium (IRDiRC), all known rare diseases (RD) should be diagnosable within a year [1]. Therefore, to wait more than one year could be considered diagnosis delay (DD). This project aims to know the time for diagnosis of people affected by a RD in Spain, the clinical determinants that increase the risk of having a DD and their psychological and social impact by comparing their situation with those in whom the diagnosis has arrived in one year or less.

**Materials and methods:** A purpose-designed form from the Spanish RD Patient Registry [2] for data-collection purposes was used. Three institutions linked to the RD collective in Spain collaborated in the study: IIER-ISCIII (research centre), FEDER (patient advocacy groups) and CREER (public administration).

**Results:** Main results were published in three open access scientific articles [3-5]:

As Figure 1 shows, 56.4% of people had experienced delay in diagnosis of their RDs, being higher in: women (OR 1.25; 95%CI 1.07–1.45); those with symptom onset at age 30–44 years (OR 1.48; 95%CI 1.19–1.84): and in mental and behavioural disorders (OR 4.21; 95%CI 2.26–7.85). The time to obtain a diagnosis and the diagnosis delay underwent a significant reduction since 1960 (*p*<0.001).

**Figure 1:**
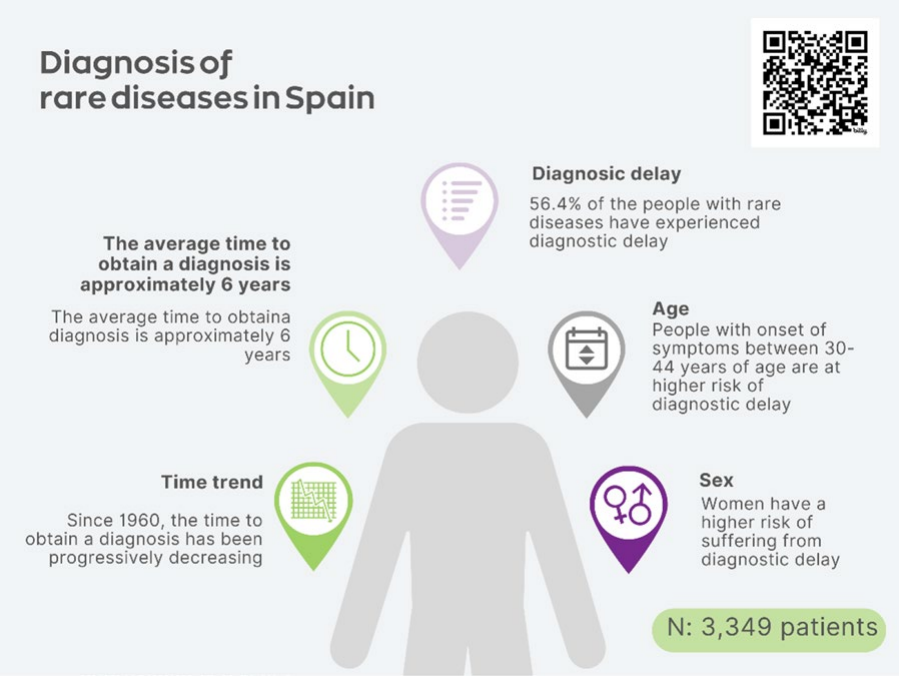
Diagnosis of rare diseases and characterization of diagnostic delay in Spain

Some of the determinants associated with experiencing DD are (Figure 2): having to travel to see a specialist other than that usually consulted in the patient’s home province (OR 2.1; 95%CI 1.6–2.9); visiting more than 10 specialists (OR 2.6; 95%CI 1.7–4.0) or being diagnosed in a region other than that of the patient’s residence (OR 2.3; 95%CI 1.5–3.6).

**Figure 2:**
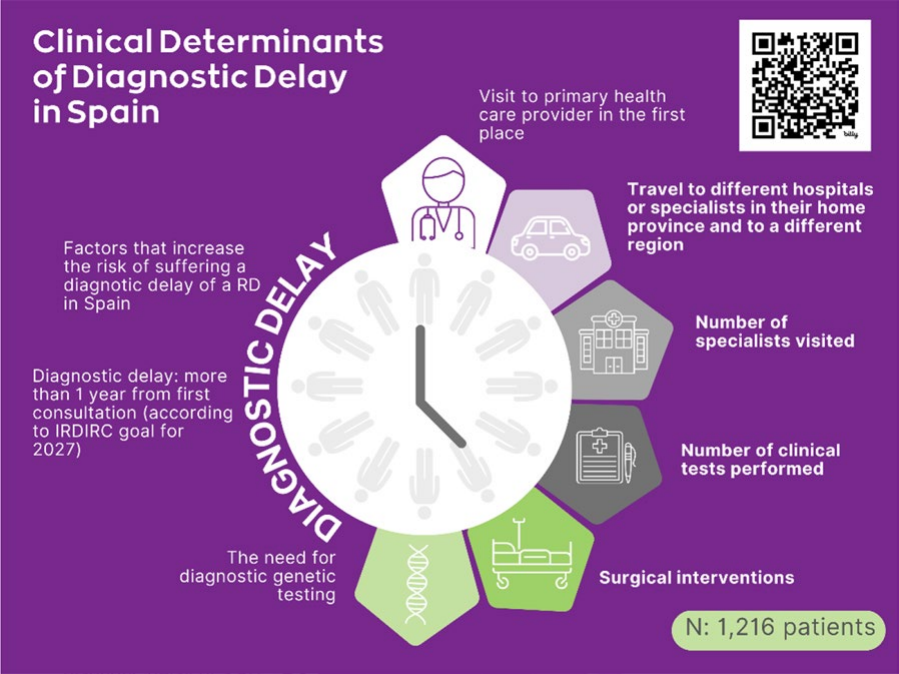
Clinical determinants of Diagnostic Delay in Spain

People who experienced DD were more in need of psychological care while searching for diagnosis (36.2% vs 23.2%; p=0.002). Reducing DD would improve psychological effects, such as concentration on everyday life (OR 3.5; 95%CI 1.5–7.9). The influence of the social implications and functional repercussions of the disease was greater in persons with DD (22.4 vs 20 and 10.6 vs 9.4, respectively). See Figure 3.

**Figure 3:**
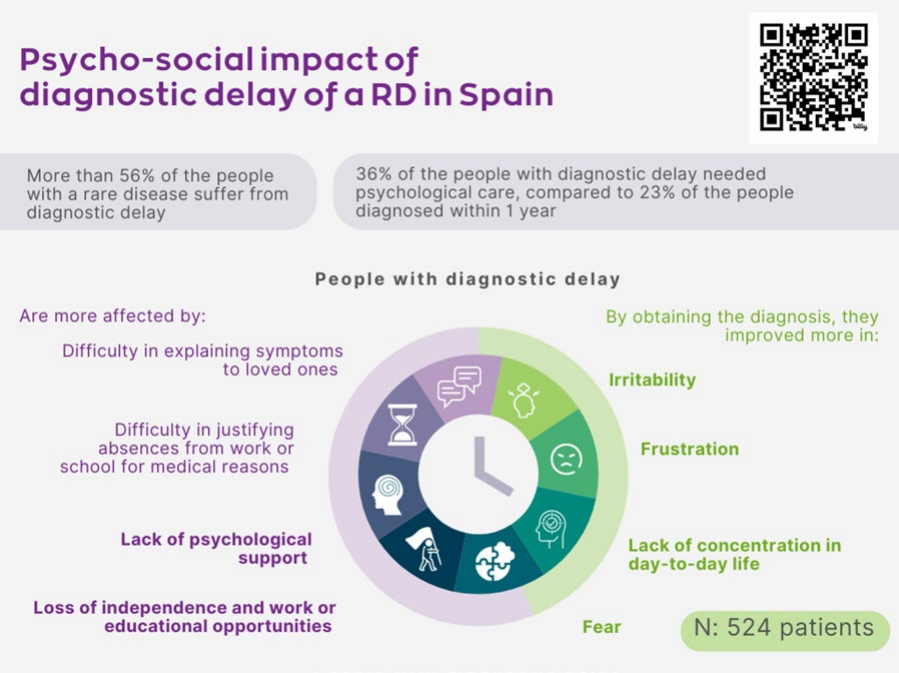
Psycho-social impact of diagnostic delay of a rare disease in Spain

**Conclusions:** These findings provide insight into the determinants that have the greatest influence on delayed diagnosis. It will also contribute to develop recommendations to reduce the time required to obtain a diagnosis, as well as correct social inequalities and improve the quality of life of people who live with a RD.

**Acknowledgements**

This research was supported by the Spanish State Research Agency, State R&D Program Oriented to the Challenges of the Society, project no. RTI2018-094035-A-I00.

**References**

- [1] Austin CP, Cutillo CM, Lau LPL, et al. Future of rare diseases research 2017–2027: an IRDiRC perspective. Clin Transl Sci. 2018;11:21–7.
- [2] Spanish Rare Diseases Patient Registry. Instituto de Salud Carlos III. Available at: https://registroraras.isciii.es. Accessed 09 May 2024.
- [3] Benito-Lozano J, Lopez-Villalba B, Arias-Merino G, Posada de la Paz M, Alonso-Ferreira V. Diagnostic delay in Rare Diseases: data from the Spanish Rare Diseases Patient Registry. Orphanet J Rare Dis. 2022;17: 418.
- [4] Benito-Lozano J, Arias-Merino G, Gómez-Martínez M, Ancochea-Díaz A, Aparicio-García A, Posada de la Paz M, Alonso-Ferreira V. Diagnostic Process in Rare Diseases: Determinants Associated with Diagnostic Delay. Int J Environ Res Public Health. 2022;19(11): 6456.
- [5] Benito-Lozano J, Arias-Merino G, Gómez-Martínez M, Arconada-López B, Ruiz-García B, Posada de la Paz M, Alonso-Ferreira, V. Psychosocial impact at the time of a rare disease diagnosis. PLoS ONE. 2023;18(7): e0288875.

## P22 Evolving experience of health care at a reference centre as reported by patients and parents of children with rare conditions over a 6 year time period

### Heather Newport, Monica Hytiris, Shannon Mullen, Arlene Smyth, Claire Dinning, Elizabeth Dougan, S. Faisal Ahmed, Martina Rodie*

#### The Office for Rare Conditions, University of Glasgow, Glasgow, UK

*Corresponding author: martina.rodie@glasgow.ac.uk

***Orphanet Journal of Rare Diseases*** 2024, 18(1): P22

**Background:** Rare conditions pose unique challenges in healthcare delivery, and feedback from patients is vital to improve care. This work aimed to follow-up on previously published data to analyse changes in patient experience at a tertiary children’s hospital from the periods of 2018–20 and 2021–23.

**Methods:** An existing questionnaire-based survey was modified to include the impact of the COVID-19 pandemic. The survey focused on information provision, patient support, satisfaction and care during the pandemic. Quantitative data from the 2018–2020 and 2021–2023 surveys underwent statistical analysis. Likert-scale data was treated as ordinal and analysed using the Mann-Whitney U-test to detect differences between survey periods. The chi-squared test was used for nominal data.

**Results:** 130 questionnaires were completed from 2018–2020 and 62 were completed from 2021-2023. 68% of patients reported they were told little or no information at diagnosis, compared with 46% prior (*p*=0.19). Current adequate understanding also changed to 53% from 66% (*p*=0.09). 72% of respondents were aware of support groups, with a previous figure of 59% (*p*=0.09), and membership changed from 55% to 67% (*p*=0.13). Overall satisfaction with the healthcare team changed from 60 to 70% (*p*=0.18). There was an increase in support for children with life-limiting conditions from 8 to 23% (*p*=0.01). In the latter survey 73% had experienced remote consultations with only 10% and 20% satisfied with video and telephone consultations respectively. Hospital appointments were cancelled in 13%, therapy appointments in 17%, investigations in 9% and surgeries in 13% of respondents during the pandemic.

**Conclusions:** This work provided insights into areas of improvement and concern. Information provision is highlighted as a concern, with a decrease in understanding since the pandemic. Addressing these areas enables healthcare providers to deliver patient-centred care and enhance quality of life. Further surveys to track changes in these benchmarks will help improve patient care.

## P23 Characteristics of patients with Type 3 Gaucher Disease (GD3) and use of medical devices and supportive services: Baseline results from the Gaucher Registry for Development, Innovation and Analysis of Neuronopathic Disease (GARDIAN)

### Suzanne Reed^1^, Joseph Milce^1^, Perrine Le Calvé^1^, Amina Omri^1^, Amal Sadou^1^, Bastien Vincent^1^, Tanya Collin-Histed^2^

#### ^1^Oracle Life Sciences, Paris, France. ^2^International Gaucher Alliance, London, United Kingdom

*Corresponding author: tanya@gaucheralliance.org

***Orphanet Journal of Rare Diseases*** 2024, 18(1): P23

**Background:** Gaucher Disease (GD) is a rare inherited metabolic disorder [1]. Type 2 (GD2) and Type 3 (GD3) are neuronopathic and cause infant death or progressive neurological deterioration. Current drug therapies do not cross the blood brain barrier and do not treat neuronopathic GD (nGD) [2]. GARDIAN is a longitudinal, global patient registry developed by the International Gaucher Alliance, patients, caregivers, clinicians, and researchers. Patient- and caregiver-reported data are collected on enzyme/genetic results, patient characteristics, neurological and non-neurological symptoms, medical history, treatment, and comorbidities. Patient-reported outcomes (PRO) and observer-reported outcomes (ObsRO) include an nGD-PRO and nGD-ObsRO to be validated within the registry. Results presented here focus on the characteristics of GD3 patients in relation to education, work, and the use of medical devices and services as reported at baseline (registry entry).

**Materials and methods:** Eligible patients or their caregivers registered and provided consent to participate. Proof of GD3 diagnosis (e.g., enzyme/genetic results or a physician’s report) was uploaded by patients/caregivers and confirmed by a dedicated medical team. Patients or their caregivers completed an online baseline questionnaire which captured data on patient characteristics, educational level, employment status, living situation, and the use of medical devices and support services.

**Results:** Baseline data were collected on 25 GD3 patients through early April 2024 as reported by patients (n=10, 40%) and parents/caregivers (n=15, 60%). Patients resided in Asia (n=10, 40%), Europe (n=10, 40%) and North America (n=5, 20%) and lived with parents/siblings (n=24, 96%) or a partner/spouse (n=1, 4%). Patients had a mean age of 15.1 years (SD: 8.4) and 48% were female. Highest level of education included early childhood/primary (32%), lower/upper secondary (40%), and Bachelor’s/Master’s (12%). Another 8% had not been to school. Patients were employed full-time (4%), part-time (12%), were on long-term disability (4%) or in other situations (e.g., still in education). Patients used various medical devices: glasses (32%), wheelchair (16%), feet orthosis (16%), back brace (12%), hearing aids (8%), walk-in shower (8%), bath lift (4%), ramps at home (4%). Patients relied on a case manager (36%), adapted school/teaching (24%), home care (20%), mental health services (16%), helpline support (12%), or institutional long-term care (12%).

**Conclusions:** GARDIAN generates valuable longitudinal, geographically diverse real-world evidence on nGD patient- and disease-related characteristics. The collection of data in a systematic and standardized manner provides a research platform for improving disease understanding, informing clinical trial design, developing safer treatments, advancing disease management, and improving patient outcomes.

**References**

- [1] Nalysnyk L, Rotella P, Simeone JC, Hamed A, Weinreb N. Gaucher disease epidemiology and natural history: a comprehensive review of the literature. Hematology. 2017 Mar;22(2):65-73.
- [2] Roshan Lal T, Sidransky E. The Spectrum of Neurological Manifestations Associated with Gaucher Disease. Diseases. 2017 Mar 2;5(1):10.

**Disclosure for all authors**

Suzanne Reed, Joseph Milce, Perrine Le Calvé, Amina Omri, Amal Sadou, and Bastien Vincent are employees of Oracle Life Sciences, France (a company that received research funds from International GARDIAN Limited), Tanya Collin-Histed is an employee of the International Gaucher Alliance.

## P24 Unveiling the epidemiological landscape of rare diseases: A demographic analysis by medical domain

### Moi Yamazaki*, Caterina Lucano, Florence Sauvage, Marc Hanauer, Valérie Serriere-Lanneau, Ana Rath

#### INSERM, US14-Orphanet, Plateforme Maladies Rares, 96 rue Didot, 75014, Paris, France

*Corresponding author:moi.yamazaki@inserm.fr

***Orphanet Journal of Rare Diseases*** 2024, 18(1): P24

**Background:** Despite recognition of rare diseases (RDs) as a public health priority, population-based studies and epidemiological data remain scarce. This scarcity poses unique challenges, such as delayed diagnosis and hindering evidence-based care. A previous study estimated a global point prevalence of RDs at 3.5–5.9% of the general population, however detailed demographics based on medical domain are less explored. [1]

**Materials and methods:** Data that was used in this analysis are publicly available through Orphadata website. (https://www.orphadata.com/orphanet-scientific-knowledge/) For this analysis, the data from the first of February 2024 was used. The epidemiological database contained data of 6,349 RDs excluding categories and subtypes of diseases, of which 3,733 are included in this analysis. After adjusting for missing data, overall mean point prevalence and its 95% confidence interval were calculated and was repeated for each medical domain.

**Results:** Among the included 3733 diseases, rare developmental defects during embryogenesis accounted for the largest single category at 37.1% (n = 1382), followed by rare neurological diseases (20.2%, n = 753) and rare inborn errors of metabolism (7.6%, n = 286). (Figure 1) The onset of RDs was most frequently observed in the neonatal period (30.7%, birth to four weeks of life), followed by infancy (25.3%, end of fourth week to 23rd month) and childhood (14.7%, 2 to 11 years). (Table 1, Figure 2) This study found that approximately 4.14% to 5.28% of the general population is affected by rare diseases, accounting for 32–40 million affected individuals in Europe, and 335–427 million worldwide, revising the figures of the previous study.1Rare developmental defects during embryogenesis is responsible for 0.77–1.32% (62–107 million, worldwide) of the population, followed by rare neurological diseases (0.39–0.75%, 32–60 million), and rare inborn errors of metabolism (0.10–0.30%, 8.1–24.3 million). (Table 2)

**Figure 1:**
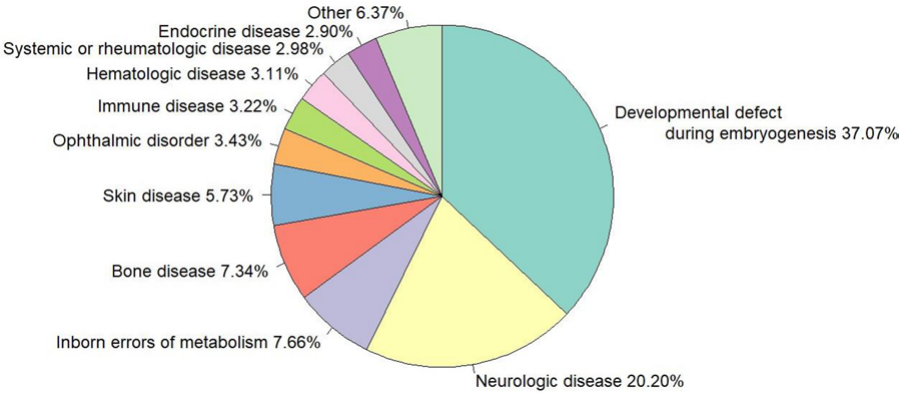
Pie chart of the percentage of RDs by medical domain (n=3724)

**Table 1:** Lower and upper limit of each age of onset category, more than one category of age of onset may be applicable to one rare disease

Age category	Lower limit	Upper limit
Neonatal	Birth	4th week of life
Infancy	The end of 4th week	23rd month of life
Childhood	2 years	11 years
Adolescence	12 years	18 years
Adult	19 years	65 years
Elderly	After 65 years	–

**Figure 2:**
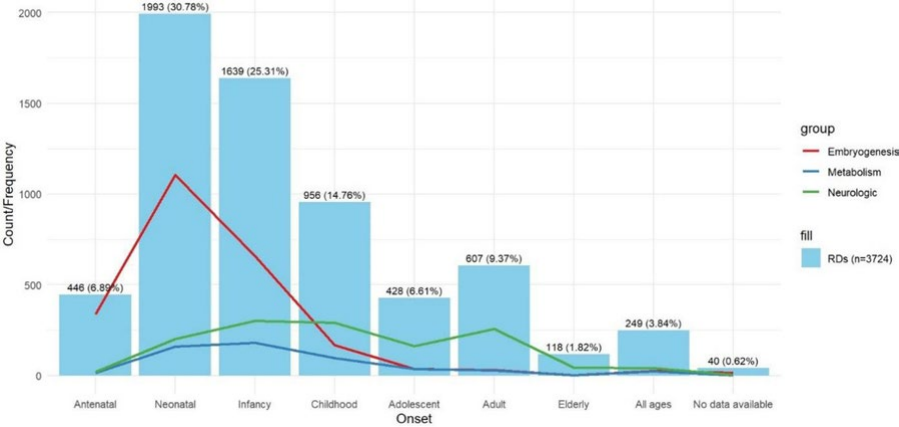
Bar plot of the onset of all RDs combined with line plot of the onset of the three most frequent domains

**Table 2:** Global point prevalence of RDs across the ten most frequent medical domains

Medical domain (number of RDs)	Minimum prevalence % (affected individual, worldwide)	Maximum prevalence % (affected individual, worldwide)
All (3724)	4.14 (335,000,000)	5.28 (427,000,000)
Rare developmental defect during embryogenesis (1382)	0.774 (62,000,000)	1.327 (107,000,000)
Rare neurological disease (753)	0.397 (32,000,000)	0.750 (60,000,000)
Rare inborn errors of metabolism (286)	0.101 (8,100,000)	0.300 (24,300,000)
Rare bone disease (274)	0.012 (983,000)	0.195 (15,800,000)
Rare skin disease (214)	0.114 (9,200,000)	0.359 (29,100,000)
Rare ophthalmic disorder (128)	0.162 (13,150,000)	0.408 (33,000,000)
Rare immune disease (120)	0.003 (246,000)	0.088 (7,100,000)
Rare hematologic disease (116)	0.151 (12,200,000)	0.471 (38,200,000)
Rare systemic or rheumatologic disease (111)	0.260 (21,000,000)	0.586 (47,400,000)
Rare endocrine disease (108)	0.117 (9,500,000)	0.404 (32,700,000)

**Conclusion:** This study indicates a narrower global point prevalence of rare diseases (4.14–5.28%) compared to the previous study (3.5–5.9%).1 Nevertheless, both ranges exhibit considerable similarity. Moreover, this study dives deeper into the point prevalence across different medical domains, a novel approach that sheds light on the complex demography of rare diseases. Despite these new insights, epidemiological data on rare diseases remain scarce and biased, reinforcing the need for stronger national surveillance systems. Additionally, the findings highlight the importance of early detection through antenatal and neonatal screening and diagnosis, particularly since nearly one-third of rare diseases manifest their initial symptoms during the neonatal period. (398 words)

**Reference**

- [1] Nguengang Wakap S, Lambert DM, Olry A, Rodwell C, Gueydan C, Lanneau V, Murphy D, Le Cam Y, Rath A. Estimating cumulative point prevalence of rare diseases: analysis of the Orphanet database. Eur J Hum Genet. 2020 Feb;28(2):165-173.

## P25 OdySMA—a quest to access: an SMA Europe advocacy tool

### Nicole Gusset^1,2^_,_ Laura Gumbert^1*^, Alice Larotonda^1^, Yasemin Erbas^1,3,4^

#### ^1^SMA Europe, Freiburg, Germany. ^2^SMA Schweiz, Heimberg, Switzerland. ^3^Spierziekten Vlaanderen, Borgerhout, Belgium. ^4^Tilburg University, Tilburg, Belgium

*Corresponding author: laura.gumbert@sma-europe.eu

***Orphanet Journal of Rare Diseases*** 2024, 18(1): P25

SMA Europe, a European umbrella of spinal muscular atrophy (SMA) patient organisations, aims to improve access to diagnosis, optimal treatment and care for all individuals living with SMA in Europe. SMA Europe’s initiative “OdySMA – a quest to access”, inspired by Homer’s epic Odyssey adventure, identifies and tracks obstacles in access to healthcare in different European countries and across the SMA spectrum. Based on the collection and analysis of more than 21 benchmark data sources, OdySMA [https://odysma.sma-europe.eu]:

1. Analyses, consolidates and makes available comparative data regarding access to diagnosis, treatment and care in all SMA Europe member countries,
2. Allows to visualise differences in access across countries and the spectrum of individuals living with SMA to identify gaps and work towards solutions,
3. Through dedicated trainings, builds capacity among patient advocates to help them build successful advocacy strategies.

OdySMA brings together systematically collected data to illustrate access pathways through interactive data visualisations. We collect “real-time” data about access to medicines and care by involving stakeholders like SMA patient organisations and the pharmaceutical industry. Our findings show persistent inequity in access to diagnosis, medicines, and care across Europe:

1. Newborn screening implementations differ in countries and by region,
2. Access to medicines varies by country and spectrum of the condition,
3. A benchmarking report on the care of adults living with SMA [https://odysma.sma-europe.eu/adult-care] identified a pressing need for updated international standards of care for this population.

Our analyses show that access to medicines and care in Europe is intricate. To exemplify the complexity of access restriction, we integrate our quantitative data visualisation tools with the stories of individuals living with SMA to show how every access limitation impacts a real life. This qualitative data sheds light on individual data-points, enhancing their relevance, making the impact of having or lacking access to diagnosis, treatment and care more concrete and tangible, and every solution more urgent and important.

A first set of these real-life stories can be found here: Real-life stories [https://odysma.sma-europe.eu/stories].

In conclusion, OdySMA is a well-established advocacy tool for our patient advocates community. OdySMA will continue to work on highlighting the identified unmet needs also in the policy environment so that gaps in healthcare systems can be closed in the future. To ensure that no one is left behind.

## P26 Highlighting the societal value of rare disease treatments could lead to improved access

### Pedro Andreu^1^, N. John Atay^2^, Enrico Piccinini^3,^ Giacomo Chiesi^3^, Gina Cioffi^4^*

#### ^1^IQVIA, Basel Switzerland, ^2^IQVIA, Munich Germany, ^3^Chiesi Farmaceutici Spa Parma Italy, ^4^Chiesi USA, Boston, MA USA

*Corresponding author: Gina.Cioffi@Chiesi.com

***Orphanet Journal of Rare Diseases*** 2024, 18(1): P26

**Aim:** We aimed to demonstrate the economic burden rare conditions impose on individuals, society, and healthcare systems to encourage policies that will lead to improved health of rare disease patients.

**Methods:** We examined direct, indirect and mortality-related [1] costs (Table 1) for 23 rare diseases (Table 2) across five therapeutic areas (Table 2) first in the United States [2] and now in France, Germany and Italy. The 23 rare diseases (Table 2) were selected from multiple sources in collaboration with Patient Advocacy Groups and others [2] and affect approximately 227,000 people in France, Germany and Italy. We benchmarked these costs against those for high-prevalence diseases including diabetes, cardiovascular disease, Alzheimer’s disease, arthritis and certain cancers. [3] We explored the burden when treatment is available and provided a scenario analysis to show what the cost would have been if there were no effective treatments available for those diseases. There is a high degree of specificity on the costs for the 23 rare diseases included in this analysis and the calculations are similar across the board in the three geographies. Information from families on their specific financial strains and attributes were not available.

**Table 1:** Cost Categories

	Direct costs	Indirect costs	Mortality costs
Definition	All direct costs related to medical care This burden is often assumed by patients and payers	All indirect costs related to a specific condition Economic burden derived mainly from patient and caregiver productivity losses	Estimated Value of Statistical Life (VSL) adjusted to patient lifespan: €5 300 000 per life in Germany €4 700 000 per life in France €3 800 000 per life to reduce the risk by one in Italy
Cost summary	Prescription drugs Medical procedures (e.g. dialysis) Hospitalization (inpatient) Hospitalization (outpatient) Home healthcare Professional services (e.g. nurse visits)	Patient productivity loss Caregiver burden Home changes Cost of secondary treatments Travelling and accommodation costs	Sum of all cash flows not generated by a patient because of an earlier-than-average death due to rare disease
Source	Patient Advocacy Groups EU health economic reports and peer-reviewed publications EU HTA reports Expert interviews (physicians, specialists and health economic experts)	EU health economic reports

**Table 2:** Summary of Selected Rare Diseases

**Metabolic disorders**	**Haematological disorders**	**Immunological disorders**	**Congenital disorders**	**Neurological disorders**
Fabry disease Gaucher disease type I Mucopolysaccharidosis (Hunter, Hurler) Ornithine transcarbamylase deficiency Phenylketonuria	Acquired aplastic anaemia Acute intermittent porphyria Atypical haemolytic uremic syndrome Beta thalassaemia major Sickle cell disease	Autoimmune encephalitis Common variable immune deficiency Juvenile idiopathic arthritis Myasthenia gravis Pemphigus vulgaris	Angelman syndrome Christianson syndrome Deletion 5p Fragile X syndrome	Amyotrophic lateral sclerosis Ataxia telangiectasia Duchenne muscular dystrophy Early onset familial Alzheimer’s disease

**Results:** The average burden for the high-prevalence diseases was €7000 per patient per year (PPPY). [3] In comparison, the average burden of the rare diseases we explored was €107 000 PPPY, an increase of more than 15 times. Of this PPPY burden, indirect costs for the 23 rare diseases average 29% of the total burden when treatment is available, rising to an average of 45% when no treatment is available. Significantly, most of the indirect costs are borne by families. We directionally estimated that without any treatment options available, the overall burden PPPY would increase by 28% across the 23 diseases in focus. (Figure 1) These data suggest that the availability of treatment creates positive value and alleviates financial strain on families and healthcare systems.

**Figure 1:**
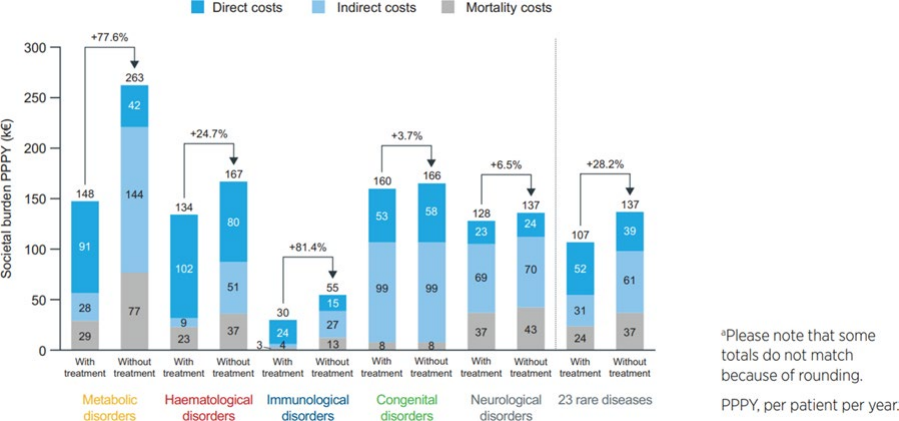
Burden of disease PPPY (k€) across selected rare diseases with and without treatment

**Conclusion:** The findings provide important insights for patients and patient representatives, as well as policymakers and other stakeholders, on the economic burden of rare conditions on individuals, society, and healthcare systems. Moreover, and our findings demonstrate the economic advantage when treatments are made available. The inequity between high-prevalence and rare diseases should be addressed at the country level and in national plans. We encourage further exploration and advocacy focused on the acute gap in research and available treatments and the totality of its impact.

**References**

- [1] Sweis NJ. Revisiting the value of a statistical life: an international approach during COVID-19. Risk Management. 2022;24:259-72.
- [2] Chiesi Global Rare Diseases. The burden of rare diseases: an economic evaluation. 2022. Available from: https://chiesirarediseases.com/assets/pdf/chiesiglobalrarediseases.whitepaper-feb.-2022_production-proof.pdf
- [3] Meersens. Chronic diseases, who will pay the bill? Available from: https://meersens.com/chronic-diseases-who-will-pay-the-bill/?lang=en

## P27 Unlocking patient data silos: creating privacy-preserving international patient registries

### Louis-Francois Arsenault, Neil Couture, Eric Faulkner, Michael Kravchyna, Aoife Manley*, Dustin O’Dell

#### SymetryML, Morristown, NJ, USA

*Corresponding author: aoife@symetryml.net

***Orphanet Journal of Rare Diseases*** 2024, 18(1): P27

**Background:** Rare diseases affect millions worldwide, yet research and development in this field are hindered by fragmented data and privacy constraints. In collaboration with a top 5 biopharma company and their global CRO, we sought to reinvent a multinational rare disease patient registry, uniting patient data from multiple countries across Europe and North America. Our goal was to enhance data access, collaboration, and analytics capabilities among key stakeholders, thereby accelerating research and regulatory evidence gathering to bring treatments to market.

**Problem:** Critical patient data essential for research and regulatory purposes were inaccessible due to stringent EU privacy regulations, leaving datasets locked in regional and national siloes. This limited opportunities for advancements in rare disease treatment and care.

**Solution:** We created a breakthrough technology, the Data Elements Matrix (DEM), to unlock and enable aggregation of sensitive patient data. The DEM is a novel data structure that completely preserves the privacy of underlying datasets (HIPPA and GDPR compliant), while retaining full analytical value of the data, enabling sharing, merging and advanced analysis. The mathematical nature of the DEM enables powerful exploratory analysis and machine learning, hyper-efficiently and cost-effectively. This technology enables secure, scalable and AI-driven solutions to drive rare disease research forwards.

**Conclusion:** As the volume of patient data and real-world evidence grows exponentially, including genetic information, accessing and uniting this data becomes increasingly critical. However, it is crucial to use this data ethically and effectively, and to preserve patient privacy. The DEM opens up a world of data collaboration, enabling us to unite and analyze patient data worldwide to accelerate the discovery and deployment of treatments and cures for rare diseases.

## P28 Centralized and up-to-date data on orphan drugs: the European Medicines Regulatory Database

### Anna MG Pasmooij^1,3^, Stefan Verweij^1^, Vincent Haverhoek^1^, Jarno Hoekman^2^, Lourens T Bloem^3^

#### ^1^Dutch Medicines Evaluation Board, Utrecht, the Netherlands. ^2^Innovation Studies, Copernicus Institute of Sustainable Development, Utrecht University, Utrecht, the Netherlands. ^3^Division of Pharmacoepidemiology and Clinical Pharmacology, Utrecht Institute for Pharmaceutical Sciences, Utrecht University, Utrecht, the Netherlands

***Orphanet Journal of Rare Diseases*** 2024, 18(1): P28

Data about drug regulation are of great value to various stakeholders such as patients, healthcare professionals, drug developers, as well as regulators themselves. However, in Europe, data about regulated drugs are dispersed over various websites and numerous documents per drug. Consequently, these data can be poorly accessed, understood, and used. We have developed the European Medicines Regulatory Database (EMRD): an up-to-date, website-based dashboard that centralizes and contextualizes data about drugs authorized and orphan designations (ODs) granted by the European Medicines Agency (EMA). The EMRD combines data that are scraped from the EMA website and the European Commission’s *Union Register of medicinal products*, including all drug labels, legal decisions, and assessment reports. Up to 31 December 2023, the EMRD’s algorithms accessed over 65,000 documents from which they extracted almost 70 variables (i.e., drug, disease, legal and regulatory characteristics) of 1716 drugs and 307 ODs. The dashboard provides explanations of these characteristics and their legal and regulatory history, as well as options to download, visualize or analyze selected data. For example, information on orphan drugs is real-time available. In the timeframe 2001–2023 (when drugs could receive OD), 1561 drugs were authorized of which 244 drugs (16%) had at least one OD at time of marketing authorization. By 31 December 2023, these 244 drugs had 307 ODs, of which most drugs had one OD (203, 83%) or two ODs (26, 11%). The highest number of ODs was six for two drugs (Glivec, Ravicti). The most common ATC codes were L:Antineoplastic and immunomodulating agents and A:Alimentary tract and metabolism. The time from designation to authorization can also be displayed with the longest time being 21 years for Xenpozyme. We will make the EMRD openly available in 2024, and are confident that it will enhance accessibility, understanding and (consistent) use of European regulatory data.

## P29 Experiences of people living with a rare disease in secondary and tertiary healthcare settings: a a scoping review

### Rita Francisco^1^, Jennifer Jones^2,3^, Michel Wensing^4,5^, Jessie Dubief^1^*

#### ^1^EURORDIS – Rare Diseases Europe, Paris, France. ^2^Genetic Alliance UK, UK. ^3^Department of Population Health Sciences, University of Leicester, UK. ^4^Heidelberg University, Heidelberg, Germany. ^5^Department of General Practice and Health Services Research, Heidelberg University Hospital, Heidelberg, Germany

*Corresponding author: jessie.dubief@eurordis.org

***Orphanet Journal of Rare Diseases*** 2024, 18(1): P29

**Background:** People living with a rare disease (PLWRD) are a heterogenous population faced with complex, and specific healthcare challenges. Patient experience is an increasingly valued healthcare quality indicator, with patient reported experience measures (PREMs) emerging as serviceable instruments (1,2)

**Material and methods:** We conducted a scoping literature review to document and map into domains and categories the published evidence on PLWRD and their carers’ experiences with secondary and tertiary healthcare. Medline, Embase, and CINAHL were searched; papers from 2005 to September 2022 were retrieved and analysed. We used the list of domains recently developed by the People Living with Chronic Conditions International Survey (PaRIS) study on healthcare experiences of people living with multiple chronic diseases (3), adapting it to allow for systematic and structured data extraction and analysis for rare diseases.

**Results:** Sixty-one papers and/or peer-reviewed abstracts were included. Table 1 lists the main characteristics of the included papers.

**Table 1:** Main characteristics of the included papers

**Paper characteristics**	**Number of papers (%)**
Study design*	
Quantitative	37 (61)
Qualitative	13 (21)
Mixed methods	6 (10)
Qualitative-quantitative	5 (8)
**Validation activities**	41 (67)
Use and/or adaptation of existing validated instruments	16 (26)
Conducted literature reviews and/or used patient experience healthcare frameworks ^a^	9 (15)
Internal consistency & other statistical tests ^b^	11 (18)
Multi-phased analysis ^c^	11 (18)
Several researchers involved in analysis and cross-checking analysis with interviewees ^c^	11 (18)
**Patient involvement **^**#**^	30 (49)
Patient participation (contribute to participant recruitment)	8 (13)
Patient engagement (review documents/instrument content, be involved in pilot testing of instruments and guides and/or in priority-setting exercises)	23 (38)
Patient involvement/partnership (being full members of advisory groups and/or partners by co-designing questions and data handling)	7 (11)
**Disease coverage**	
Single disease	35 (57)
Multi-disease	26 (43)
**Geographic coverage**	
Single country	44 (72)
Multi-country	19 (31)
**Target groups**	
Only patient-reported experiences	26 (43)
Only proxy-reported experiences	11 (18)
Both patient and proxy-reported experiences	24 (39)

Informed by published evidence, we propose a rare disease patient healthcare experience definition with 11 domains and 71 categories. The most represented domains are ‘self-management’ (89%, 54/61), ‘patient-centredness’ (87%, 53/61), and ‘comprehensiveness’ (80%, 50/61); whereas the least mentioned are ‘safety’ (13%, 8/61), ‘trust’ (23%, 14/61) and ‘service aspects of care’ (33%, 20/61). In between sit ‘coordination’ (72%,44/61), ‘access’ and ‘overall perceived quality of care’ (69%,42/61 each), ‘continuity’ (57%,35/61) and ‘diagnosis & treatment/interventions’ (54%, 33/61) (Figure 1). Forty-two questionnaires were described in the selected papers: 12 are validated instruments with eight of them having been specifically developed and/or validated for rare diseases. Only one instrument, the Cystic Fibrosis Patient Experience and Satisfaction with Care Services, covers all domains and the highest number of categories (31/71).

**Figure 1:**
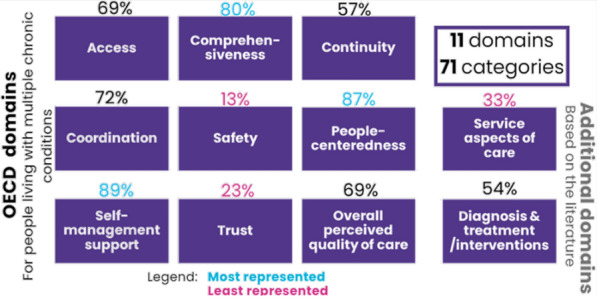
Preliminary definition of rare disease patient experience with care.

**Conclusions:** This study outlines rare disease patient experience with secondary and tertiary care from a patient / carer perspective, putting forward a preliminary definition of what healthcare experience for PLWRD encompasses. Consulting with diverse rare disease community groups is key to further frame, refine and validate our findings.

**Acknowledgments**

The authors are grateful to the members of the Scientific Working Group for their expert advice and involvement in this work.

**References**

- [1] Wolf JA, Niederhauser V, Marshburn D, LaVela SL. Defining Patient Experience. Patient Exp J [Internet]. 2014 [cited 2023 Dec 13];1(1):7–19. Available from: https://pxjournal.org/journal/vol1/iss1/3
- [2] Oben P. Understanding the Patient Experience: A Conceptual Framework. J Patient Exp [Internet]. 2020 Dec [cited 2023 Dec 13];7(6):906–10. Available from: https://www.ncbi.nlm.nih.gov/pmc/articles/PMC7786717/
- [3] De Boer D, Van Den Berg M, Ballester M, Bloemeke J, Boerma W, De Bienassis K, et al. Assessing the outcomes and experiences of care from the perspective of people living with chronic conditions, to support countries in developing people-centred policies and practices: study protocol of the International Survey of People Living with Chronic Conditions (PaRIS survey). BMJ Open [Internet]. 2022 [cited 2023 Dec 13];12(e061424.). Available from: http://bmjopen.bmj.com/
- [4] Badger S, Barendrecht S, Bros-Facer V, de Graaf J, Desir-Parseille J, Kaisler R, et al. Short guide on Patient Partnerships in Rare Disease Research Projects [Internet]. 2021 [cited 2024 May 30]. Available from: https://www.ejprarediseases.org/wp-content/uploads/2021/03/SHORT-GUIDE-ON-PATIENT-PARTNERSHIPS-IN-RARE-DISEASE-RESEARCH-PROJECTS.pdf

## P30 Third French National Plan for Rare Diseases: a diagnosis observatory overview

### Sarah Otmani^1^, Arnaud Sandrin^1^, BNDMR operational Unit^1^, Anne-Sophie Lapointe^2^, Vincent Vauchel^2,^ Céline Angin^1^

#### ^1^French national rare disease registry (BNDMR), AP-HP, Paris, France. ^2^French Ministry for Labor, Health, and Solidarity, General Directorate of Health Care Provider (DGOS), Paris, France

*Corresponding author: contact.bndmr.nck@aphp.fr

***Orphanet Journal of Rare Diseases*** 2024, 18(1): P30

Reducing the diagnostic delay to obtain a correct and precise diagnosis was one of the French National Rare Disease (RD) Plan 3 (PNMR3) [https://solidarites-sante.gouv.fr/IMG/pdf/pnmr3_-en.pdf] priorities (action 1.7), funded by the French Ministry of Health to the tune of 3 M€ per year for the duration of the plan (2018-2023).

To monitor the diagnostic delay evolution and measure the impact of actions carried out, a diagnostic observatory [https://solidarites-sante.gouv.fr/soins-et-maladies/prises-en-charge-specialisees/maladies-rares/article/l-observatoire-du-diagnostic] was set up, based on indicators from the French National Rare Disease Registry (BNDMR) [https://www.bndmr.fr/]. Indeed, French RD expert centers must collect in the BNDMR and for every RD patient a minimal data set (MDS) [https://www.bndmr.fr/les-donnees-collectees/le-set-de-donnees-minimal/] that includes level of diagnostic assertion, age at diagnosis, etc. This observatory aims to ensure consistency of practices, share inspiring initiatives and take into account recent discoveries in medical care.

Several strategies to reduce the diagnostic delay were deployed. The main one was to reassess undiagnosed patient files in light of recent scientific advances and new test opportunities; in three years, more than 100,000 patient records were reviewed. A significant number of people were recruited (more than 300 over 3 years, especially clinical research assistants and technicians) that allowed to increase the quality and completion of data in the BNDMR. Various initiatives were promoted by the RD reference centers (CRMR) and patient organisations (200 documents created and 141 revised, 1400 people trained, research projects funded...). Finally, 5 dedicated data collections were created in the BNDMR to precisely describe the patient’s diagnostic odyssey [https://www.bndmr.fr/les-donnees-collectees/recueils-complementaires-eid/] and the MDS was expanded with a distinction between clinical and genetic diagnosis, deeper genetic tests precisions and genomic anomalies description.

Those actions helped undiagnosed patients to access new diagnostic investigations and enabled the identification of patients without diagnosis who could be prioritized for genomic analysis. This work will be amplified during the next National Rare Disease Plan 4 starting in 2024 [https://sante.gouv.fr/actualites/presse/communiques-de-presse/article/vers-un-4eme-plan-national-maladies-rares-pnmr4-catherine-vautrin-sylvie]

## P31 Unifying voices: creating a European Alliance to advocate for a better and longer life for people living with Alpha-1

### Fernanda Aspilche Ferro^1,2^, Heinz Stutzenberger^1,3^, Elena Goyanes Vilar^1,4^, Karen O’Hara^1,5^, Frank Willersinn^1,2*^, Cristina Barbiero^1,6^, Marion Wilkens^1,3^, Renate Shashoua^1,7^, Veronica Lopez Gousset^8^

#### ^1^Alpha-1 Europe Alliance asbl, Brussels, Belgium. ^2^Alpha-1 Plus Belgium asbl, Brussels, Belgium. ^3^Alpha1 Deutschland, Gernsheim, Germany. ^4^Asociación Alfa-1 de España, Santiago de Compostela, Spain. ^5^Alpha-1 UK Support Group, Droitwich, United Kingdom. ^6^Alfa1-AT National Association, Sarezzo, Italy. ^7^Alpha-1 Schweiz, Zug, Switzerland. ^8^VLG Global, Paris, France, 75011

*Corresponding author: info@alpha1europe.org

***Orphanet Journal of Rare Diseases*** 2024, 18(1): P31

**Objectives:** Over the past year, leaders of alpha-1 antitrypsin deficiency (alpha-1) patient organizations across the European Region have united forces to elevate the voice of the patient community at the European level. Rare disease communities, like that in alpha-1, must learn from each other and join efforts to raise awareness, foster research, and advocate for access to timely diagnosis, individualized therapies, and holistic care.

**Methods:** Starting January 2023, three workstreams were created to agree on remit and working principles of the Alliance, set up practical processes and tools, and create a framework for sustainable success. Activities were split across the workstreams as follows: (1) developed the organization’s external image, including name, mission, vision, and visual identity; (2) defined strategy and objectives, including specific projects, timelines, and budgets; (3) established registration country, articles of incorporation, and registered the Alliance. See Table 1 for more details on the objectives across each workstream. For each objective, workstream members developed rationale and recommendations that would then be shared with all national group leader representatives volunteering across the workstreams. Proposals were then voted on to come to decisions in a democratic way. Thirteen representatives from national groups in Austria, Belgium, Germany, Ireland, Italy, Norway, Spain, Switzerland, and the United Kingdom volunteered across the workstreams to establish the Alliance.

**Table 1:** Workstream objectives to support the establishment of the Alpha-1 Europe Alliance

Workstream 1	Workstream 2	Workstream 3
Propose official name Define mission and vision Define visual identity Guide website creation	Draft project plan with short- and longer-term objectives, measures for success and corresponding budget Define organizational structure and growth plan based on project plan Establish membership program (types, benefits, fees, etc.)	Select organizational seat / registration country Guide development of Statutes, including definition of governance structure Guide development of start-up contracts and develop other governance tools (by-laws, rules for Alliance, etc.) Register organization in local jurisdiction

**Results:** As of 17 October 2023, the Alpha-1 Europe Alliance [https://alpha1europe.org], based in Belgium, is established and operational and as of 27 November 2023 counts on 11 members across the European community.

**Conclusions:** Collaborating as an Alliance, the alpha-1 patient community will engage in policy discussions to improve access and foster research toward a cure, ensuring patients’ involvement in research and clinical development. The Alliance will undertake campaigns to familiarize the European population with the challenges of living with alpha-1 and support member organizations in building their reach and capacity.

The process of creating an alliance may help other communities join forces at the regional level. Key learnings included choosing a registration country with a mature patient organization but also with low-level of formalities for the registration process and the establishment of a bank account, the advantages of hiring a project manager to coordinate patient leader volunteers, and the importance of involving the wider community periodically along the process to ensure the Alliance is reflective of the community’s needs and expectations.

**Acknowledgements**

The Alpha-1 Europe Alliance would like to thank all patient group leaders and committed individuals that contributed to the establishment of the Alliance, including Stilla Beitz (Austria), Geraldine Kelly (Ireland), Knut Skaar (Norway), Sabine Bucher (Switzerland), Tanya Jones (United Kingdom) and Lucía García Sidera (Spain).

## P32 Beyond a single primary endpoint: optimizing research in rare disease using multiple prioritized outcomes

### Jean-Christophe Chiêm^1^*, Samuel Salvaggio^1^, Everardo Saad^2^, Vaïva Deltuvaite-Thomas^2^, Mickaël De Backer^1^, Marc Buyse^1,2,3^

#### ^1^One2Treat SA, Louvain-la-Neuve, Belgium. ^2^International Drug Development Institute, Louvain-la-Neuve, Belgium. ^3^Hasselt University, Hasselt, Belgium

*Corresponding author: jean-christophe.chiem@one2treat.com

***Orphanet Journal of Rare Diseases*** 2024, 18(1): P32

Organizing randomized clinical trials in the context of rare diseases faces many challenges: recruitment is high-stakes, patient populations are heterogeneous, and clinical symptoms can be multifaceted. Meanwhile, the standard evaluation of clinical trials is based on a single primary outcome that is considered clinically relevant and statistically sensitive for detecting a difference between experimental treatment and standard of care. Consequently, the use of information on additional outcomes that could be particularly crucial for patients and clinicians is suboptimal.

The statistical method of Generalized Pairwise Comparisons (GPC) is a recent proposal that allows the simultaneous evaluation of multiple outcomes of interest [1]. These multiple outcomes can be prioritized to reflect an order of clinical relevance or analyzed simultaneously with a weighting scheme, which leads to a consistent combination. A threshold of clinical relevance can also be applied to translate a discriminant difference for each outcome.

Using GPC, we were able to drastically reduce the required sample size of a clinical trial in Sanfilippo A syndrome by considering five outcomes while preserving adequate statistical power to show a relevant effect of a novel therapeutic approach compared with the data from natural history [2].

In other settings, GPC has allowed incorporating patients’ opinions regarding their perceived clinical importance of various outcomes. This interactive exercise of eliciting patient preferences can assist in designing trials that address the true unmet needs of the patients and better reflect clinical reality.

Through prioritization of multiple outcomes, GPC can also deliver a precise quantitative assessment of the benefit-risk balance and account for correlations between them. The results can then offer robust empirical evidence of one therapy's efficacy and safety profile compared to other treatments. Benefit-risk considerations are a crucial component in guiding comprehensive cost-effectiveness evaluations, leading to transparent reimbursement decisions.

With increasing interest from authorities (FDA, EMA), GPC can play a primary role in optimizing clinical trials for rare diseases beyond the study of a single primary endpoint by capturing all outcomes that matter. Moreover, by offering a platform for patient engagement and participation in the trial design, the method paves the way for the future of patient-centric medicine.

**References**

- [1] Buyse, M. Generalized pairwise comparisons of prioritized outcomes in the two-sample problem. Stat. Med. 2010, 29, 3245–3257.
- [2] Deltuvaite-Thomas V., De Backer M., Parker S. et al. Generalized pairwise comparisons of prioritized outcomes are a powerful and patient-centric analysis of multi-domain scores. Orphanet J Rare Dis 2023, 18, 321.

## P33 The role of patient participation in the German NARSE (National Registry for Rare Diseases) and the accompanying evaluation project FAIR4Rare

### Claudia Finis*

#### Berlin Institute of Health at Charité, Berlin, Germany

*Corresponding author: claudia.finis@bih-charite.de

***Orphanet Journal of Rare Diseases*** 2024, 18(1): P33

**NARSE:** The National Registry for Rare Diseases (NARSE) is funded by the Eva Luise and Horst Köhler Foundation (ELHKS). It is intended to enable

- The consent-based entry of patient data by the treating physicians
- The findability and networking of patients
- Entries in the NARSE registry by patients themselves or patient organisations
- For the first time, a comprehensive overview of all rare disease patients living in Germany
- Exchange of data at European and international level

The aim of NARSE is to record as many affected people as possible in the long term to determine the health effects and care structures of rare diseases in Germany. Already during the development of NARSE, the evaluation by patients led to changes in the NARSE.

In the pilot phase, the focus is on three rare diseases of which one is Osteogenesis Imperfecta (OI) represented by the German OI organisation (DOIG). The aim is to revise the minimum data set, i.e. the absolutely necessary information about a disease, for the registry. The expert knowledge of patients plays an essential role to improve the quality of the data.

**FAIR4Rare:** The FAIR4Rare evaluation project is funded by the Innovationfonds of the

Federal Joint Committee and will run for a period of 30 months. Project partners from 12 University hospitals, 3 patient organisations and the ELHKS are examining the extent to which NARSE is accepted by users and what further developments are necessary to meet the special needs of patients with rare diseases.

The patient organisations evaluate NARSE in regard to the needs of people with a rare diseases in general, but also in regard to disease-specific issues. As a project partner, DOIG is planning further involvement of its members for the project and the satellite Documentation through presentations, consultations and workshops.

**Satellite Documentation for OI:** The NARSE basically contains the set of common data elements for rare diseases registration. These data are not sufficient to conduct diagnosis-specific research. Satellite documentation is planned for this purpose. It is currently being set up under the leadership of the author, who is herself a combined patient, patient representative and scientist.

As a European Patient Advocate at European registry for rare bone and mineral conditions and member of the study group of the disease-specific module for OI, the author has decided to include it in the satellite registry. Furthermore, it covers items from German OI experts and items patient experts consider important.

## P34 Benchmark of phenotypic driven similarity methods and patients’ clinical signs annotations exploration

### Maroua Chahdil^1,4^* , Caterina Lucano^1^ , Carolina Fabrizzi^1^ , Leslie Matalonga^2^ , Anaïs Baudot^3^ , Marc Hanauer^1^ , Ana Rath^1^ , David Lagorce^1^ , Laurent Tichit^4^

#### ^1^US14 - Orphanet Plateforme Maladies Rares, Paris, France. ^2^CNAG-CRG, Centre for Genomic Regulation (CRG), The Barcelona Institute of Science and Technology, Barcelona, Spain. ^3^Aix Marseille Univ, INSERM, MMG, Marseille, France. ^4^Aix Marseille Univ, CNRS, I2M, Marseille, France

*Corresponding author: Maroua.chahdil@inserm.fr

***Orphanet Journal of Rare Diseases*** 2024, 18(1): P34

**Background:** Rare diseases (RD) have a prevalence of no more than 1/2000 of the European population and are characterised by the difficulty of obtaining a correct and timely diagnosis. According to Orphanet, 72.5% of RDs are of genetic origin, although 35% of them do not yet have an identified causative gene. In addition, a significant proportion of patients with suspected genetic RD receive inconclusive exome/genome sequencing [1]. As a follow up of a previously published methodology to prioritise variants for reanalysis based on phenotypic similarities [2], we aimed at benchmarking phenotypic similarity methods and to understand variations on phenotypic annotations.

**Materials and methods:** We used a data model of known RDs annotated with genetic information and phenotypes (Human Phenotype Ontology (HPO) terms), from the Orphanet RD Knowledge base [3], and patients’ cases retrieved during the the Solve-RD project [4,5], a project aiming to identify the molecular causes of undiagnosed RD. The methodology relied on the Resnik similarity measure [6] , which calculates information content (IC). In order to enhance the performance of our approach, we refined Resnik and added Lin computation [7]. We developed OrphaScape, a user-friendly tool to visualise similarity results between cases and RDs, aiming at guiding variant prioritisation. We design a benchmark to assess the suitability of the similarity measures for our data. We perform a cumulative rank distribution analysis of cases associated with diseases based on clinical signs.

**Results:** It was observed that more than half of the associations are retrieved with a good rank using Resnik which is the most appropriate for our data. In addition, solved cases-RD associations based on clinical signs were reviewed by a physician at Orphanet, who evaluated the relevance of each proposed diagnosis. A qualitative and quantitative exploration was performed to better understand patients’ HPO profiles. Qualitatively, we observed differences on the use of HPO terms by the European Reference Networks (ERNs) involved in SolveRD which could introduce some bias. However, from a quantitative perspective, there is no discrepancy among phenotypic annotations: the average depth of HPO terms did not exhibit any significant variation.

**Conclusions:** Our results can raise awareness among clinicians on the quality of phenotypic annotations, to help reduce human bias. In conclusion, the Resnik measure is the most appropriate for our data. This study shows the importance of phenotypic quality annotation to improve diagnostic hypotheses based on phenotypic similarity computations, while also promoting the most suitable similarity measure for this type of data. Acknowledgement The Solve-RD project has received funding from the European Union’s Horizon 2020 research and innovation programme under grant agreement No 779257.

**References**

- [1] Nguengang Wakap S, Lambert DM, Olry A, Rodwell C, Gueydan C, Lanneau V, et al. Estimating cumulative point prevalence of rare diseases: analysis of the Orphanet database. Eur J Hum Genet. 2020 Feb;28(2):165–73.
- [2] Lagorce D, Lebreton E, Matalonga L, Hongnat O, Chahdil M, Piscia D, et al. Phenotypic similarity-based approach for variant prioritization for unsolved rare disease: a preliminary methodological report. Eur J Hum Genet. 2024 Feb;32(2):182–9.
- [3] Orphadata – Orphanet datasets [Internet]. [cited 2024 May 30]. Available from: https://www.orphadata.com/
- [4] Matalonga L, Hernández-Ferrer C, Piscia D, Solve-RD SNV-indel working group, Cohen E, Cuesta I, et al. Solving patients with rare diseases through programmatic reanalysis of genome-phenome data. Eur J Hum Genet. 2021 Sep;29(9):1337–47.
- [5] Zurek B, Ellwanger K, Vissers LELM, Schüle R, Synofzik M, Töpf A, et al. Solve-RD: systematic pan-European data sharing and collaborative analysis to solve rare diseases. Eur J Hum Genet. 2021 Sep;29(9):1325–31.
- [6] Lin D. An Information-Theoretic Definition of Similarity. In: Proceedings of the Fifteenth International Conference on Machine Learning. San Francisco, CA, USA: Morgan Kaufmann Publishers Inc.; 1998. p. 296–304. (ICML ’98).
- [7] Resnik P. Using Information Content to Evaluate Semantic Similarity in a Taxonomy [Internet]. arXiv; 1995 [cited 2023 Jun 2]. Available from: http://arxiv.org/abs/cmp-lg/9511007

## P35 Novel budget impact model for DMD

### Natalia Arrabal^1^, Marco Ratti^1^, Valérie Deroo^1^

#### Value & Access, Italfarmaco group, Italy

***Orphanet Journal of Rare Diseases*** 2024, 18(1): P35

**Background:** Duchenne Muscular Dystrophy (DMD) is a rare, progressive, lethal X-linked recessive disorder, caused by the lack of dystrophin protein that lead to a sequence of pathological events (muscle fiber injury, activation of chronic inflammatory pathways, impairment of muscle regeneration mechanisms, fibrogenesis and adipogenesis) resulting in impaired and loss of upper limb function, loss of ambulation and complications that significantly impact patients’ quality of life^1-5^. The loss of ambulation is the key milestone in the depletion of HRQoL in DMD patients. There is a high unmet need for new and effective treatments which demonstrate a clinically meaningful improvement in the treatment of DMD patients compared with the current standard of care (SoC)^6^. In most European countries, reimbursement decision for new treatments rely on budget impact analysis (BIA). This study aims to develop a dynamic budget impact model in DMD, to evaluate the economic impact of delaying loss of ambulation (LoA).

**Materials and Methods:** A targeted literature review was conducted to identify previous BIM in DMD. Clinical experts and health economists were consulted to validate model structure, and data inputs, to reflect current clinical practice.

**Results:** No BIM in DMD were identified. A Markov model was developed to simulate the BI of a new treatment in DMD. The model first estimates patient’s number based on DMD prevalence and incidence. The model then simulates DMD patients’ distribution from ambulatory, non-ambulatory, and death health states with and without DMD treatment to estimate the economic implications of delaying loss of ambulation. Costs in scope are drug costs, and direct and indirect costs associated to ambulatory and non-ambulatory phase.

**Conclusions:** Future BIM for new DMD treatments with the potential to delay loss of ambulation, should consider not only the cost associated to the introduction of a new treatment, but also the full economic impact of delaying LoA including the social perspective.

**References**

- [1] Ryder, S., Leadley, R.M., Armstrong, N., et al. "The burden, epidemiology, costs and treatment for Duchenne muscular dystrophy: an evidence review." Orphanet J Rare Dis. 2017; 12(1): 79
- [2] Bez Batti Angulski, A., Hosny, N., Cohen, H., et al. "Duchenne muscular dystrophy: disease mechanism and therapeutic strategies." Front Physiol. 2023; 14: 1183101.
- [3] Consalvi, S., Saccone, V., Giordani, L., et al. (2011). "Histone deacetylase inhibitors in the treatment of muscular dystrophies: epigenetic drugs for genetic diseases." Mol Med. 2011; 17(5-6): 457-465.
- [4] Giuliani, G., Rosina, M., Reggio, A. "Signaling pathways regulating the fate of fibro/adipogenic progenitors (FAPs) in skeletal muscle regeneration and disease." Febs j. 2022; 289(21): 6484-6517.
- [5] Sandonà, M., Cavioli, G., Renzini, A., et al. "Histone Deacetylases: Molecular Mechanisms and Therapeutic Implications for Muscular Dystrophies." Int J Mol Sci. 2023; 24(5).
- [6] Andreozzi, V., Labisa, P., Mota, M., et al. "Quality of life and informal care burden associated with duchenne muscular dystrophy in Portugal: the COIDUCH study." Health Qual Life Outcomes. 2022; 20(1): 36.

## P36 Development of the Atlas of Clinical Syndromes: an advanced tool for enhancing the diagnosis and treatment of rare diseases

### Christopher Schippers^1^*, Julia Sellin^1,2^, Rupert Conrad^3^, Martin Mücke^1,2^

#### ^1^Center for Rare Diseases, Medical Faculty, RWTH Aachen University, Aachen, Germany. ^2^Department of Digitalization and General Practice, Medical Faculty, RWTH Aachen University, Aachen, Germany. ^3^Department of Psychosomatic Medicine and Psychotherapy, University Hospital Münster, Münster, Germany

*Corresponding author: cschippers@ukaachen.de

***Orphanet Journal of Rare Diseases*** 2024, 18(1): 36

**Background:** The effective diagnosis and treatment of rare diseases (RD) represent significant challenges due to their low prevalence and the complexity of their symptoms. To address these challenges, the "Atlas of Clinical Syndromes" is being developed, aiming to provide physicians with a rapid and efficient overview of more than 400 syndromes associated with RDs. This initiative is designed to complement existing resources such as Orphanet [www.orpha.net] and the German SE-Atlas [www.se-atlas.de] by covering the most prevalent rare syndromic diseases as identified by Orphanet. The atlas aims to become the successor to the widely acclaimed "Leiber, The Clinical Syndromes" receiving substantial interest from the medical community (Figure 1).

**Figure 1:**
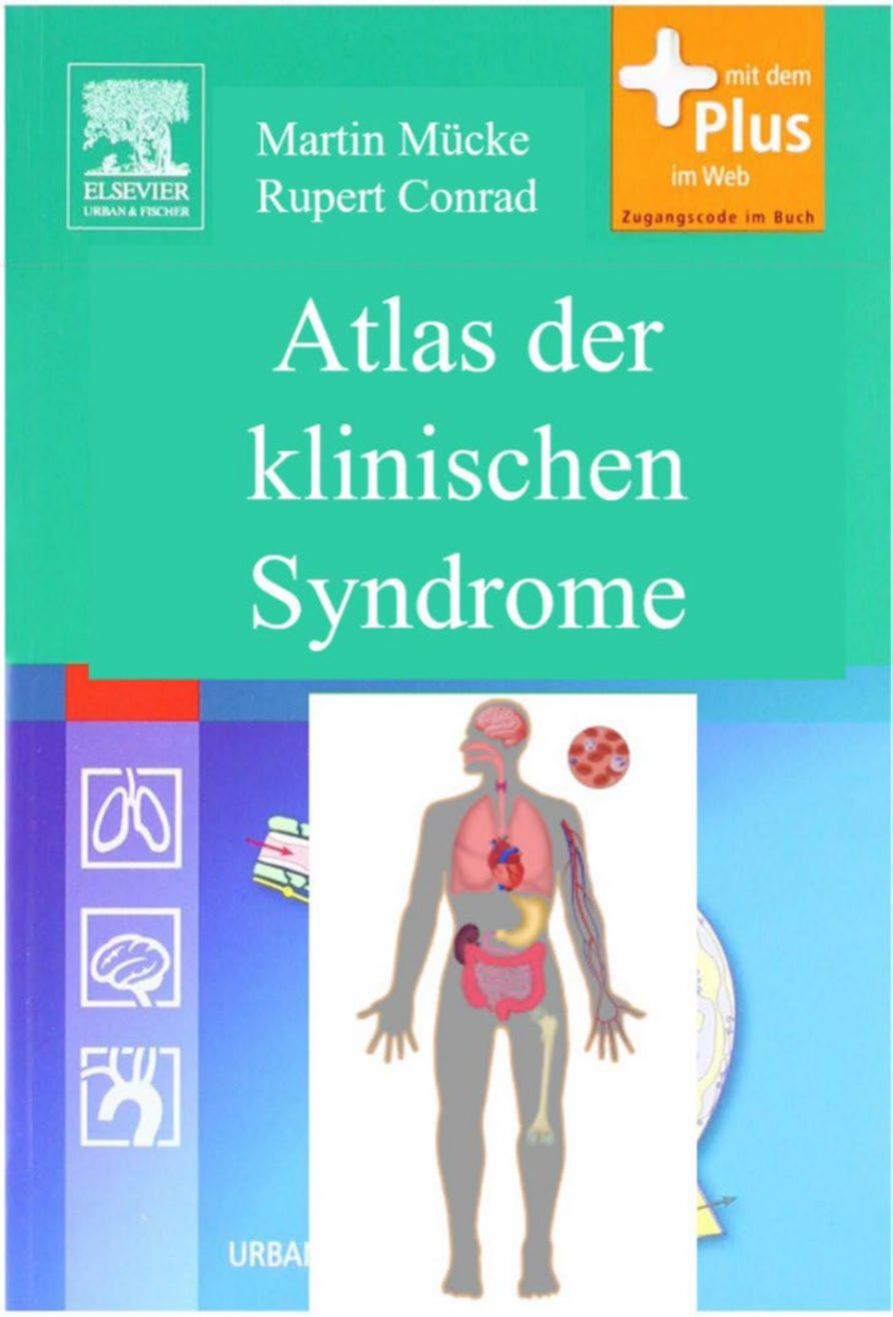
Draft of the cover page of the Atlas of Clinical Syndromes

**Layout and Content:** In an innovative approach, the atlas will be available in both print and online versions, with the latter being regularly updated to incorporate the latest advancements in the field. The language will be English.

Each syndrome will be presented on a double page, featuring essential data including the orphacode, synonyms, a comprehensive definition, symptoms, diagnostic criteria, etiology, pathogenesis, treatment options, prognosis, and additional resources such as relevant links and illustrative images (Figures 2 and 3).

**Figure 2:**
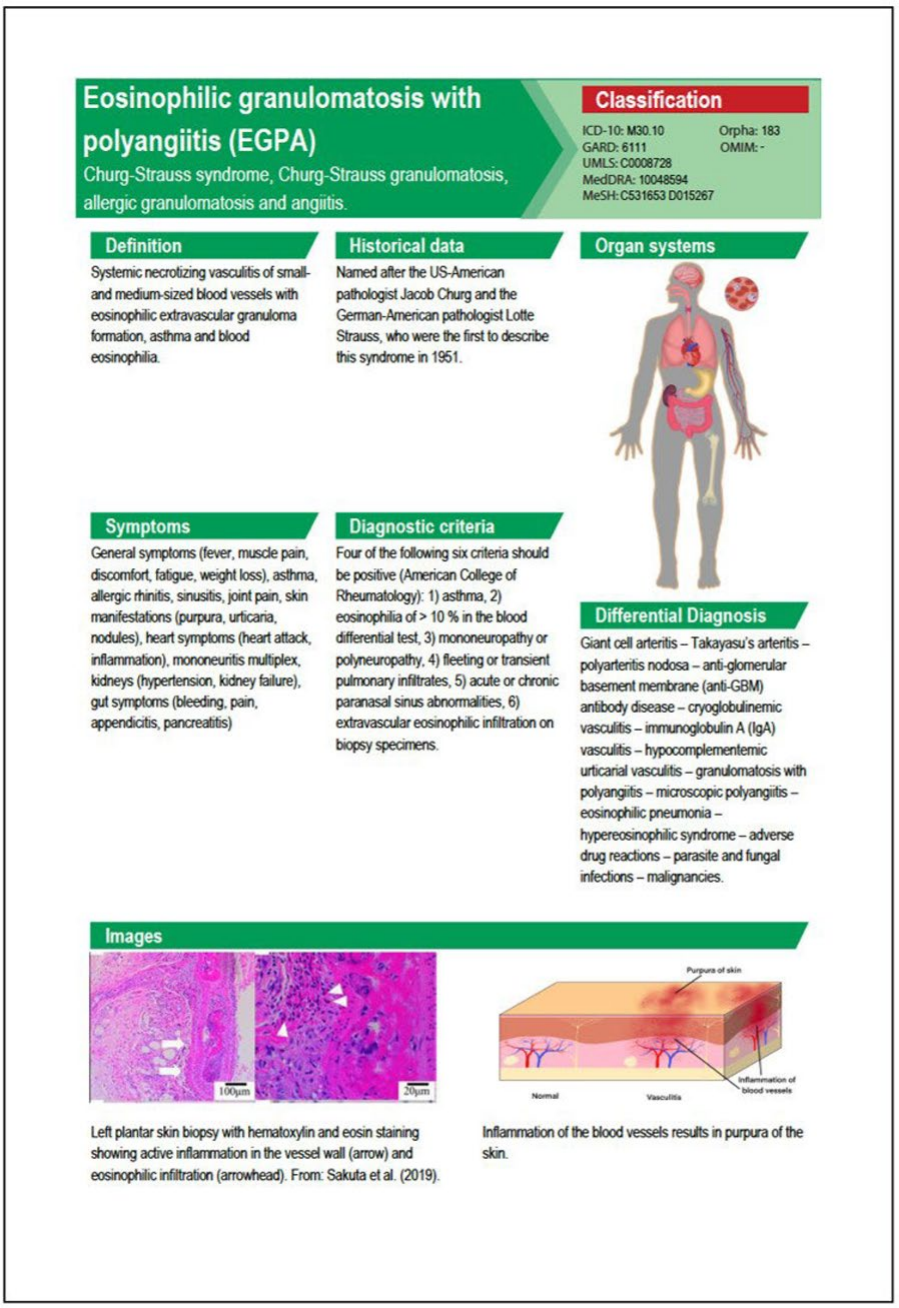
Example of a description of a rare disease on a double-page spread, left page

**Figure 3:**
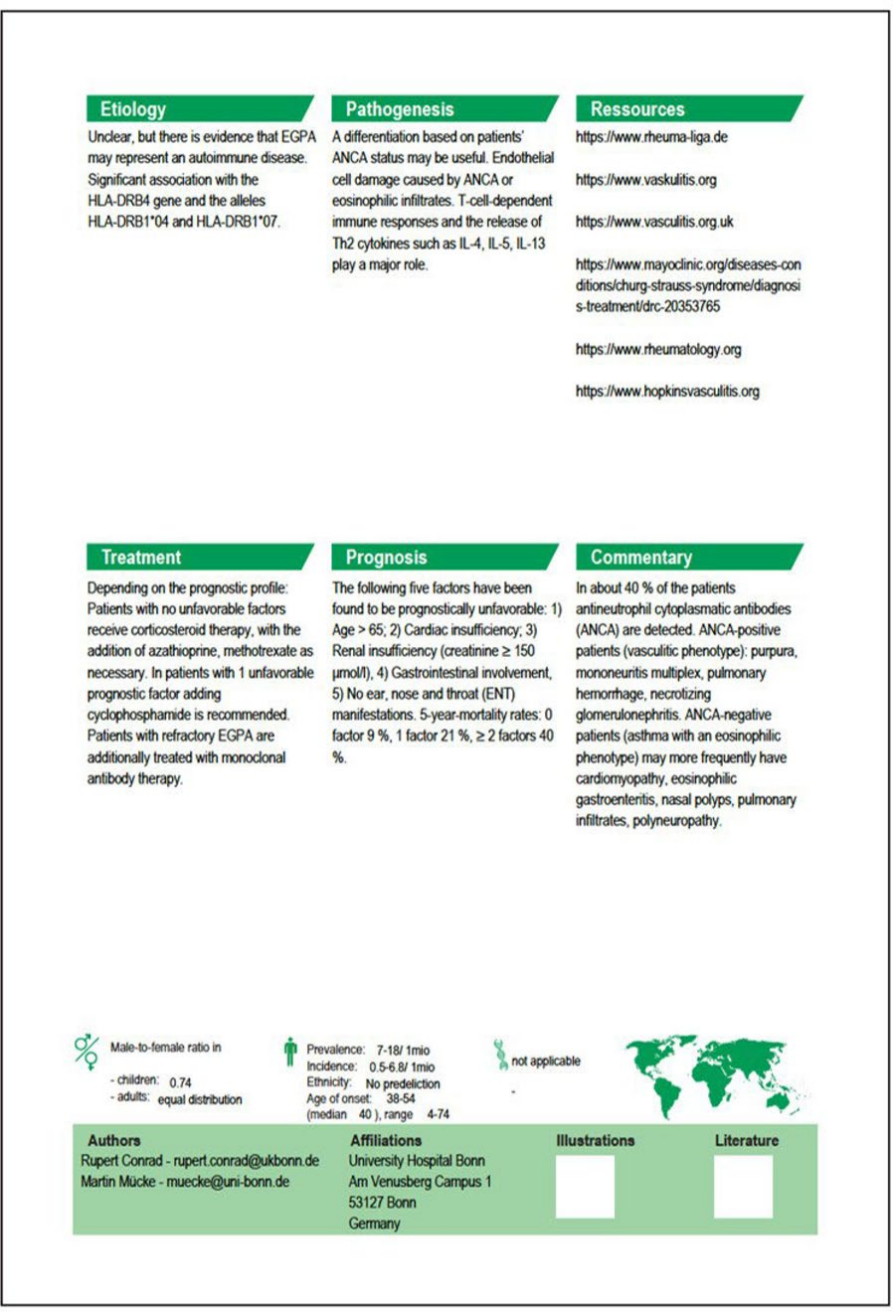
Example of a description of a rare disease on a double-page spread, right page

**World-wide cooperation:** The project has garnered the support of over 400 international experts in RDs, spanning from Boston to Seoul, highlighting the global commitment to improve outcomes for patients with rare conditions. Furthermore, collaboration with patient organizations is underway, ensuring that the atlas reflects the needs and experiences of those directly affected by RDs.

**Advantages:** The atlas has potential to:

- revolutionize the diagnostic process
- facilitate early intervention
- improve the quality of care for patients with RDs

Through this collaborative effort, the atlas seeks to empower healthcare professionals and patient representatives with a ‘state-of-the-art’ resource that bridges the gap in knowledge and fosters a more inclusive and informed medical community.

**Contact:** This is a living project to which we invite all interested experts – be they healthcare professionals or patient representatives – to contribute.

Please contact us at the e-mail address provided if you are interested.

## P37 Project proposal: Variability in Human Phenotype Ontology encoding and implications for rare disease

### Janina Schönberger^1^*, Jean Tori Pantel^2,3^*, Christopher Schippers^3^, Peter N Robinson^4^, Dominik Seelow^1^

#### ^1^Exploratory Diagnostic Sciences, Berlin Institute of Health @ Charité, Berlin, Germany. ^2^Department of Digitalization and General Practice, Medical Faculty, RWTH Aachen University, Aachen, Germany. ^3^Center for Rare Diseases, Medical Faculty, RWTH Aachen University, Aachen, Germany. ^4^ Medical Health Data Sciences, Berlin Institute of Health @ Charité, Berlin, Germany

*Corresponding author: janina.schoenberger@bih-charite.de; jepantel@ukaachen.de

***Orphanet Journal of Rare Diseases*** 2024, 18(1): P37

**Background:** The Human Phenotype Ontology [https://hpo.jax.org] (HPO) is a globally established standard for documenting human phenotypes. Figure 1 illustrates some of the many uses of the HPO which include interoperability and reproducible research. Its main aim is to enable reliable descriptions of clinical signs and symptoms. One use case is the computer-aided analysis of phenotypic anomalies to support the diagnosis of patients with an unclear diagnosis or a suspicion of a rare disease.

**Figure 1:**
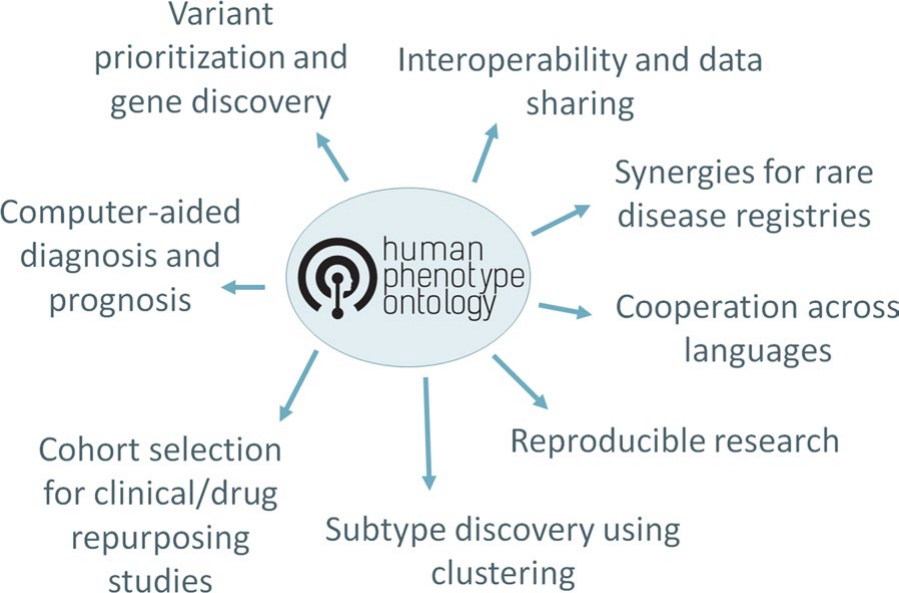
Advantages of standardized phenotyping with the Human Phenotype Ontology.

However, the effectiveness of these diagnostic support algorithms is influenced by the subjective evaluation of the examiner. Despite the uniformity of the HPO standard, notable variability exists in its practical application, particularly regarding symptom recognition and translation of phenotypic traits into HPO terms. Possible variations include the hierarchy level and the number of terms chosen, as illustrated in Figure 2 and Table 1. Clinical practice shows that the number of selected HPO terms and the selected hierarchy level cumulatively influence the results of diagnostic support systems. Vice versa, objective and unbiased descriptions of patients’ phenotypes are required to improve the accuracy of diagnostic decision support systems.

**Figure 2:**
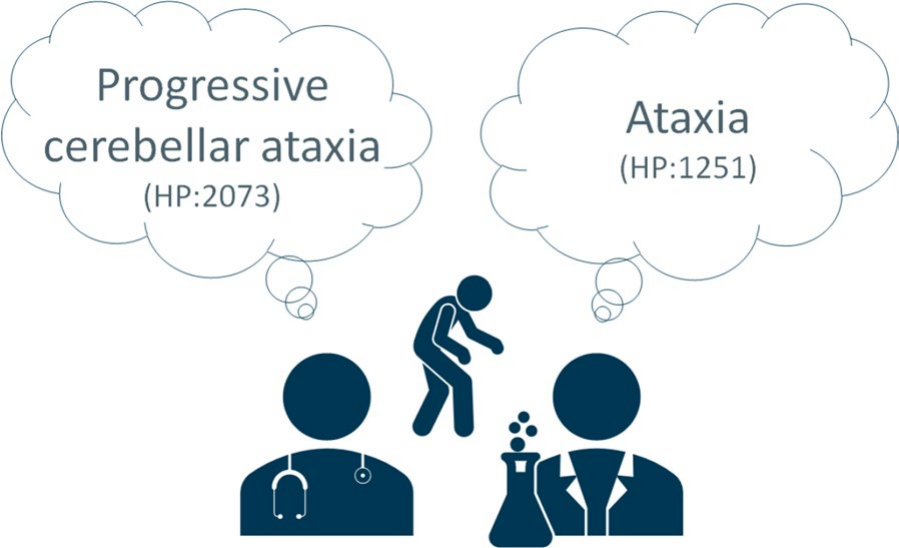
Symbolic image to illustrate the subjectivity of phenotyping with the Human Phenotype Ontology.

**Table 1:** An anecdotal example of two physicians assigning different terms from the Human Phenotype Ontology to the same patient

Physician A		Physician B	
HP:0001251	Ataxia	HP:0002073	Progressive cerebellar ataxia
HP:0002191	Progressive spasticity	HP:0001257	Spasticity
HP:0001272	Cerebellar atrophy	HP:0002352	Leukoencephalopathy

**Methodology:** Recognizing the impact of these variations, our project systematically evaluates patient phenotype annotations by different physicians. To explore the variability between phenotypes recorded by different physicians, we will ask physicians from different specialties and with different experience levels to record the phenotypes of patients using the HPO.

In a preliminary pilot phase, we will use text descriptions of patients suffering from rare diseases, followed by a second phase in which multiple physicians see and phenotype the same patients. We will not focus on single diseases but include patients from different medical specialties.

Inter-rater agreement between physicians will be calculated using semantic similarity measures that consider the hierarchical structure of the HPO. Aggregated results will be published, and feedback will be collected to investigate how the user-friendliness of the HPO may be improved.

**Community involvement:** We are collaborating with a growing number of medical centers, doctors, patients, and patient organizations. We invite everyone interested in contributing to our project to contact the corresponding authors.

## P38 The EJP RD Virtual Platform: building a network of rare disease data resources

### Ana Rath^1*^, Franz Schaefer^2^, Sergi Beltran^3^, Marco Roos^4^, Anthony Brookes^5^, Chris Evelo^6^, Friederike Ehrhart^6^ on behalf of EJP RD Pillar 2 partners

#### ^1^Inserm, US14 – Orphanet. Plateforme Maladies Rares. 96, rue Didot 75014 Paris, France. ana.rath@inserm.fr. ^2^Center for Pediatrics and Adolescent Medicine, Heidelberg University,INF 430, 69120 Heidelberg, Germany. franz.schaefer@med.uni-heidelberg.de. ^3^ Centro Nacional de Análisis Genómico (CNAG), C/Baldiri Reixac 4, 08028 Barcelona, Spain sergi.beltran@cnag.eu. ^4^Department of Human Genetics, Leiden University Medical Center, Leiden, The Netherlands m.roos@lumc.nl.^5^ Department of Genetics and Genome Biology, University of Leicester, University Road, Leicester, United Kingdom ajb97@leicester.ac.uk. ^6^ Department of Bioinformatics – BiGCaT, NUTRIM/MHeNs, Maastricht University, Universiteitssingel 60, 6229 ER Maastricht, The Netherlands. Chris.evelo@maastrichtuniversity.nl, friederike.ehrhart@maastrichtuniversity.nl

***Orphanet Journal of Rare Diseases*** 2024, 18(1): P38

**Introduction:** Addressing the rare disease (RD) challenges of accelerating diagnosis and therapeutics development requires improving the efficiency of conducting research. Researchers need to have easy access to a variety of resources and data ready to accelerate their research. Surveys of the RD community in 2019 showed that key data resources relevant to RD research in Europe are in a state of fragmentation, lack of interoperability, and that RD researchers were largely unaware of them​. The EJP RD Virtual Platform (VP) aims at creating a modular ecosystem of resources empowered to foster RD research.

**Methods:** The EJP RD VP developed the architecture, standards, methodologies and tools for a federated network of resources to be discovered via various querying tools​​. The VP enables 3 levels of connection ranging from basic discovery to federated analysis. To join the network, resources such as patient registries, biobanks, cell and mice model catalogues, genomic data repositories and others are supported by documentation and tools and assisted by a team of data stewards in implementing a common set of specifications and requirements to increase their FAIRness. Resources remain in full control of their content and connection level and of the information they want to expose. Feedback from network resources and end-users is continuously used to enhance the VP.

**Results:** Two versions of the VP were released between August 2023 and February 2024. Resources were guided in the onboarding process, resulting in 23 resources discoverable, including catalogues, registries, biobanks, knowledge bases, omics repositories, etc, of which 6 currently allow record-level querying. An online portal [1] was created to enable end-users to interact with this network, conduct searches by disease or gene, and sort results by various demographic filters​. A test-bed for advanced federated querying and analysis is under development, where use cases are currently piloted across multiple data resources.

**Conclusion:** The EJP RD VP has set common standards, methodologies and tools now implemented by resources, forming an ecosystem ready for efficient RD research. Participating resources are now enhanced for RD research. Next steps include upscaling the VP in the number, variety and depth of connection of data resources, and offering training to end-users and resources to enrich their experience in exploiting the VP’s functionalities.

**Acknowledgment**

The EJP RD initiative has received funding from the European Union’s Horizon 2020 research and innovation programme under grant agreement N°825575

**Reference**

- [1] https://vp.ejprarediseases.org

## P39 Privacy-preserving linkage of multi-modal pseudonymised rare disease data

### Dieter Hayn*, Emanuel Sandner, Karl Kreiner, Guenter Schreier

#### Center for Health & Bioresources, AIT Austrian Institute of Technology GmbH, Graz, Austria

*Corresponding author: dieter.hayn@ait.ac.at

***Orphanet Journal of Rare Diseases*** 2024, 18(1): P39

In rare diseases, it is paramount to fully exploit all available data, which are distributed over several multimodal resources, including biomaterials, images, clinical trials, registries, and many others. Many of these resources are pseudonymised and (as required by the General Data Protection Regulation GDPR [1]), different pseudonyms are used in different resources. This makes it hard to link such records without registering specific patients twice. With Privacy-Preserving Record Linkage (PPRL), personalised or pseudonymised datasets can be joined, without disclosing the patients’ identities (Figure 1). PPRL is a key technology for rare disease research [2].

**Figure 1:**
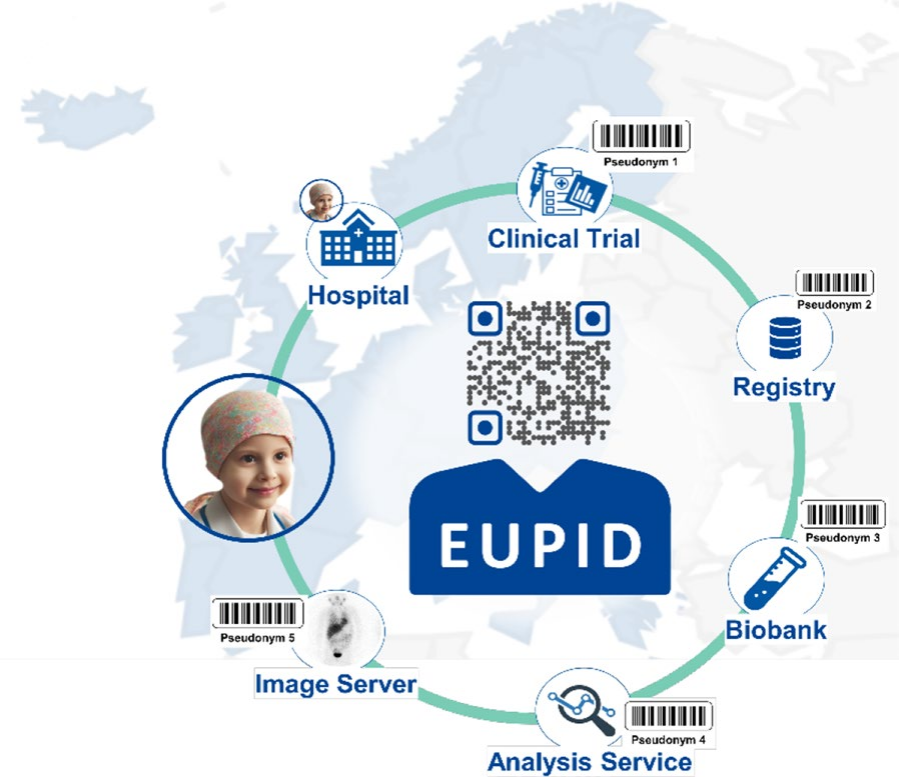
Privacy-Preserving Record Linkage (PPRL) Services such as EUPID can be used to link multi-modal personalised and pseudonymised data sources, such as clinical trials, registries, biobanks, analysis services or image servers, while ensuring the patient’s privacy.

There is a huge number of use cases for PPRL in rare diseases, which derive from six key dimensions to specify use cases: (1) Different types of distributed personalised records; (2) Pseudonymisation; (3) Different types of distributed pseudonymised records; (4) Linkage of the (personalised and/or pseudonymised) records; (5) Temporary or persistent linked data repositories; (6) Different kinds of data analysis, from simple counting to artificial intelligence. PPRL can be used for primary use (e.g., pseudonymised virtual tumour boards with multi-centric experts) and secondary use scenarios (e.g., retrospective linkage of biobanking data with multiple clinical trial databases).

The EUPID Services [3] represent a PPRL Service, which is well-proven, GDPR-compliant, approved by an external security audit. They have been used widely for rare disease research in primary and secondary use scenarios (see e.g., [4-7]). By state-of-the-art encryption and hashing algorithms, the EUPID Services support privacy-preserving linkage of distributed records. Optionally, they also support a specific, structured protocol for re-identification of patients in emergency situations by one or more trusted third parties. The EUPID Services have been applied on prospectively collected data, and on retrospective datasets.

In conclusion, PPRL Services represent a valuable technology for GDPR-compliant, multi-centric and/or multi-modal project data management, long-term registries, and various other rare disease scenarios.

**References**

- [1] THE EUROPEAN PARLIAMENT AND OF THE COUNCIL. REGULATION (EU) 2016/679 OF THE EUROPEAN PARLIAMENT AND OF THE COUNCIL of 27 April 2016 on the protection of natural persons with regard to the processing of personal data and on the free movement of such data, and repealing Directive 95/46/EC (General Data Protection Regulation). 2016.
- [2] Vassal G, Lazarov D, Rizzari C, Szczepański T, Ladenstein R, Kearns PR. The impact of the EU General Data Protection Regulation on childhood cancer research in Europe. Lancet Oncol. 2022;23(8):974-5.
- [3] Nitzlnader M, Schreier G. Patient identity management for secondary use of biomedical research data in a distributed computing environment. Stud Health Technol Inform. 2014;198:211-8.
- [4] Hayn D, Falgenhauer M, Kropf M, Nitzlnader M, Welte S, Ebner H, et al. IT Infrastructure for Merging Data from Different Clinical Trials and Across Independent Research Networks. Stud Health Technol Inform. 2016;228:287-91.
- [5] Martí-Bonmatí L, Alberich-Bayarri Á, Ladenstein R, Blanquer I, Segrelles JD, Cerdá-Alberich L, et al. PRIMAGE project: predictive in silico multiscale analytics to support childhood cancer personalised evaluation empowered by imaging biomarkers. Eur Radiol Exp. 2020;4(1):22.
- [6] Ebner H, Hayn D, Falgenhauer M, Nitzlnader M, Schleiermacher G, Haupt R, et al. Piloting the European Unified Patient Identity Management (EUPID) Concept to Facilitate Secondary Use of Neuroblastoma Data from Clinical Trials and Biobanking. Stud Health Technol Inform. 2016;223:31-8.
- [7] Haupt R, Essiaf S, Dellacasa C, Ronckers CM, Caruso S, Sugden E, et al. The 'Survivorship Passport' for childhood cancer survivors. Eur J Cancer. 2018;102:69-81.

